# The First Co-Opted Endogenous Foamy Viruses and the Evolutionary History of Reptilian Foamy Viruses

**DOI:** 10.3390/v11070641

**Published:** 2019-07-12

**Authors:** Pakorn Aiewsakun, Peter Simmonds, Aris Katzourakis

**Affiliations:** 1 Department of Microbiology, Faculty of Science, Mahidol University, Bangkok 10400, Thailand; 2 Nuffield Department of Medicine, University of Oxford, South Parks Road, Oxford OX1 3SY, UK; 3 Department of Zoology, University of Oxford, South Parks Road, Oxford OX1 3SY, UK

**Keywords:** foamy virus, spumavirus, reptile foamy virus, endogenous foamy virus, endogenous retrovirus, ancient retroviruses, co-evolution, co-speciation, foamy virus-host interactions

## Abstract

A recent study reported the discovery of an endogenous reptilian foamy virus (FV), termed ERV-Spuma-Spu, found in the genome of tuatara. Here, we report two novel reptilian foamy viruses also identified as endogenous FVs (EFVs) in the genomes of panther gecko (ERV-Spuma-Ppi) and Schlegel’s Japanese gecko (ERV-Spuma-Gja). Their presence indicates that FVs are capable of infecting reptiles in addition to mammals, amphibians, and fish. Numerous copies of full length ERV-Spuma-Spu elements were found in the tuatara genome littered with in-frame stop codons and transposable elements, suggesting that they are indeed endogenous and are not functional. ERV-Spuma-Ppi and ERV-Spuma-Gja, on the other hand, consist solely of a foamy virus-like *env* gene. Examination of host flanking sequences revealed that they are orthologous, and despite being more than 96 million years old, their *env* reading frames are fully coding competent with evidence for strong purifying selection to maintain expression and for them likely being transcriptionally active. These make them the oldest EFVs discovered thus far and the first documented EFVs that may have been co-opted for potential cellular functions. Phylogenetic analyses revealed a complex virus–host co-evolutionary history and cross-species transmission routes of ancient FVs.

## 1. Introduction

Foamy viruses (FV; the *Spumaretrovirinae* subfamily) are a unique subgroup of retroviruses (family *Retroviridae*) comprising an independent lineage basal to all other exogenous retroviruses [1]. FV surveillance and the discovery of their endogenous retrovirus (ERV) counterparts revealed that the host range of FVs covers a wide range of vertebrates, including mammals [2,3], amphibians [4], lobe-finned fish [5], bony fish [4,6], and cartilaginous fish [4,7], considerably wider than those of other retrovirus groups. Owing to the wealth of sequence data and the identification of multiple instances of endogenization for FVs, the longer-term evolutionary history of FVs can be investigated in great detail. For example, analysis of endogenous and modern-day viral sequences revealed that FVs have been broadly co-diversifying with their hosts since the origin of vertebrates, dating back almost half a billion years ago to the early Palaeozoic Era [4]. 

A recent study reported the discovery of the first reptilian endogenous FV (EFV) in the tuatara genome, namely ERV-Spuma-Spu [8]. Phylogenetic analysis showed that ERV-Spuma-Spu is basal to the clade of mammalian FVs. Based on this finding, the authors speculated that the reptilian FV lineage may have diverged from the mammalian FV linage more than 320 million years ago under the virus–host co-speciation assumption. Nevertheless, since there was effectively only one reptilian FV linage in the study, the inferred co-speciation could not be verified, and a history of viral cross-species transmissions might have been overlooked. Indeed, this was shown to be the case before for the lobe-finned fish EFV, CoeEFV [4].

Here, we further characterize ERV-Spuma-Spu and report two additional reptilian EFVs found in the genomes of panther gecko (*Paroedura picta*) and Schlegel’s Japanese gecko (*Gekko japonicus*), designated ERV-Spuma-Ppi and ERV-Spuma-Gja, respectively. Evolutionary analyses together with other currently available FV and EFV sequences suggest that these reptile EFVs do not form a monophyletic clade and that they are significantly younger than their hosts. This in turn suggests that, in contrast to what was previously suggested, their ancestors likely originated from cross-species transmissions, where one gave rise to ERV-Spuma-Spu and the other gave rise to the two gecko EFVs. Our results improve our understanding of how FVs evolved and interacted with their hosts in the distant past.

## 2. Materials and Methods

### 2.1. ERV-Spuma-Spu Mining

The tuatara genome (*Sphenodon punctatus*; accession number: QEPC01000000) was searched using tBLASTn and a CoeEFV Pol protein query with an E-value cut off of 1 × 10^−6^. This returned 20,581 hits from 2370 contigs (Table S1). These hits were then combined together (including the sequence between the two hits) if they were ≤ 5000 base pairs apart or overlapping and were in the same orientation. This resulted in 12,520 merged Pol hits (Table S2).

These hits were subsequently subjected to reciprocal BLASTx against a database of retrovirus proteins one by one with an E-value cut off of 1 × 10^−6^ (Table S2). If the best match protein did not belong to a member of the *Spumaretrovirinae* subfamily or the *Spumavirus* genus, the hit was then excluded from the downstream analyses. Ultimately, 87,387 retrovirus proteins were retrieved from the National Center for Biotechnology Information (NCBI) protein database on the 7^th^ of June 2018 using 3 search queries. The first query was ‘txid11632[Organism:exp] NOT “partial” AND 500:100000[SLEN]’, which retrieved proteins that belong to the members of the *Retroviridae* family [NCBI: txid11632] with a length between 500 and 100,000 amino acids and were not annotated as partial (86,662 sequences). The second query was ‘txid186534[Organism:exp] NOT “partial” AND 500:100000[SLEN]’, which retrieved proteins that belong to the members of the *Caulimoviridae* family [NCBI: txid186534] with a length between 500 and 100,000 amino acids and were not annotated as partial (720 sequences). The last query was ‘txid186665[Organism:exp]’, which retrieved proteins that belong to the members of the *Metaviridae* family [NCBI: txid186665] (5 sequences). Out of 12,520 Pol hits, only 2757 exhibited the greatest similarity to FV proteins (Table S2). The rest were removed from the subsequent dataset.

We noted that some of these 2757 Pol sequence candidates, nevertheless, may actually have been those of Class III ERVs and not actually of EFVs. To further exclude false positive hits, we used them as queries in a BLASTx search against 194 retrovirus and ERV Pol protein sequences, publicly available from the database held at http://bioinformatics.cvr.ac.uk/paleovirology/site/html/retroviruses.html. Those with the best hit protein that did not belong to the *Spumavirus* genus at an E-value cut off of 1 × 10^−6^ were further excluded from the dataset. Only 1959 sequence candidates remained after this procedure (Table S2).

To recover potentially full elements, we extracted these 1959 Pol hits from the tuatara genome with 10,000 base pairs extended on both ends. They were then searched against the CoeEFV Env protein query using tBLASTn. The analysis showed that only 165 out of 1959 sequences exhibited similarity to CoeEFV Env protein at the 1 × 10^−6^ E-value cut off (Table S3). These 165 endogenous viral elements were designated ERV-Spuma-Spu elements.

### 2.2. Consensus Sequence Reconstruction

**Figure 1:**
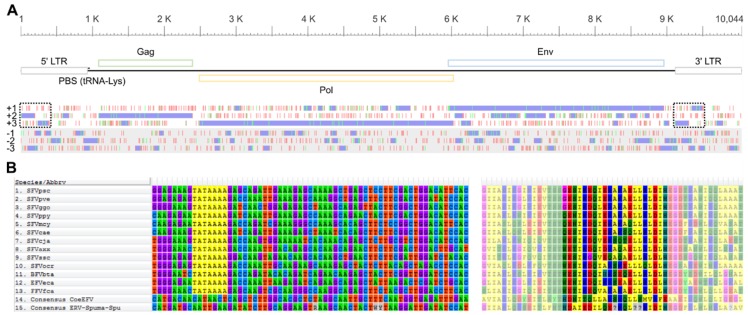
Reconstructed, putative, ERV-Spuma-Spu genome (**A**) and foamy virus (FV) internal promoters (**B**). Top-A, scale bar indicates nucleotide position. Middle-A, schematic diagram representing the genomic organisation of ERV-Spuma-Spu. LTR: long terminal repeat (grey); PBS: primer binging site; Gag: *group antigen* gene (green); Pol: *polymerase* gene (yellow); Env: *envelope* gene (blue). Bottom-A, start (green) and stop (red) codon positions in the six translation frames (+1, +2, +3, −1, −2, and −3). Potential open reading frames are shown in purple. The dotted boxes indicate the two open reading frames identified as a single accessory gene in [8]. The nucleotide sequences of consensus ERV-Spuma-Spu can be found in Figure S1 and Data S3. Left-B, internal promoters (TATAAAA) towards the 3’ end of the *env* gene could be identified in all mammalian FVs (highlighted in yellow) but were absent from CoeEFV (endogenous FVs) and ERV-Spuma-Spu. Right-B, protein sequences used to guide the nucleotide alignment. Those corresponding to the sequences on the left are shown in brighter colors.

The top 20 elements of the 165 ERV-Spuma-Spu elements that exhibited the greatest similarity to the concatenated CoeEFV Pol-Env protein sequence were aligned and used to reconstruct a consensus sequence. At the time of analysis, the quality of the tuatara genome was low, however, containing many large strings of undetermined nucleotides (‘N’s). Furthermore, the majority of the identified ERV-Spuma-Spu elements were also interrupted by transposable elements. Because of this, the standard protocol of consensus sequence reconstruction (or ancestral sequence reconstruction) inferred false gaps where they should not have been. To overcome this problem, we only allowed gaps in the consensus sequence if there were more than 15 sequences containing gaps in that particular position; otherwise, a consensus base pair from non-gap sequences would be inferred. Standard ambiguous bases were used in the case of base count ties. The consensus sequence of the virus internal region was inferred separately from the LTR portion (Data S1), and the consensus LTR sequence was inferred from both 5’ and 3’ LTRs (Data S2). Only 16 LTR sequences from 11 elements could be aligned with confidence. ORFfinder (https://www.ncbi.nlm.nih.gov/orffinder/) was used to identify open reading frames, and tRNAScan (http://lowelab.ucsc.edu/tRNAscan-SE/) was used to identify the primer binding site. We also attempted to identify the internal promoter in ERV-Spuma-Spu by comparing its sequence to those of mammalian FVs. The consensus sequence is in Figure 1, Figure S1, and Data S3. As previously reported [8], reciprocal BLASTp searches showed that the proteins identified from the consensus sequence were most similar to those of modern-day FVs, supporting that these ERV-Spuma-Spu elements are indeed FVs.

### 2.3. EFVs in Other Reptiles

Pol and Env protein sequences of the consensus ERV-Spuma-Spu and CoeEFV were used in a tBLASTn search against the NCBI Whole Genome Shotgun database restricted to reptiles and excluding the tuatara genome to examine if other reptile genomes had any EFVs.

Numerous tBLASTn hits were returned from six reptile genomes showing similarity to the Pol protein sequences. From each genome, we selected one to five top hits (19 hits in total) and examined if they were most similar to modern-day FVs. Reciprocal BLASTx analysis suggested that none of them were FV-like, however (Table S4). We thus did not analyze these sequences any further.

In contrast, only two FV-like *env* elements were found. One was found in the genome of the panther gecko (*Paroedura picta*; BDOT01000314.1:c548029-545198), and the other was found in the genome of Schlegel’s Japanese gecko (*Gekko japonicus*; LNDG01066615.1:c46188-43360). Neither elements contained in-frame stop codons or transposable elements. Results from reciprocal BLASTx searchers (Table S4) suggested that they were indeed EFVs, showing the greatest similarity to modern-day FVs. They were designated ERV-Spuma-Ppi and ERV-Spuma-Gja, respectively.

**Figure 2:**
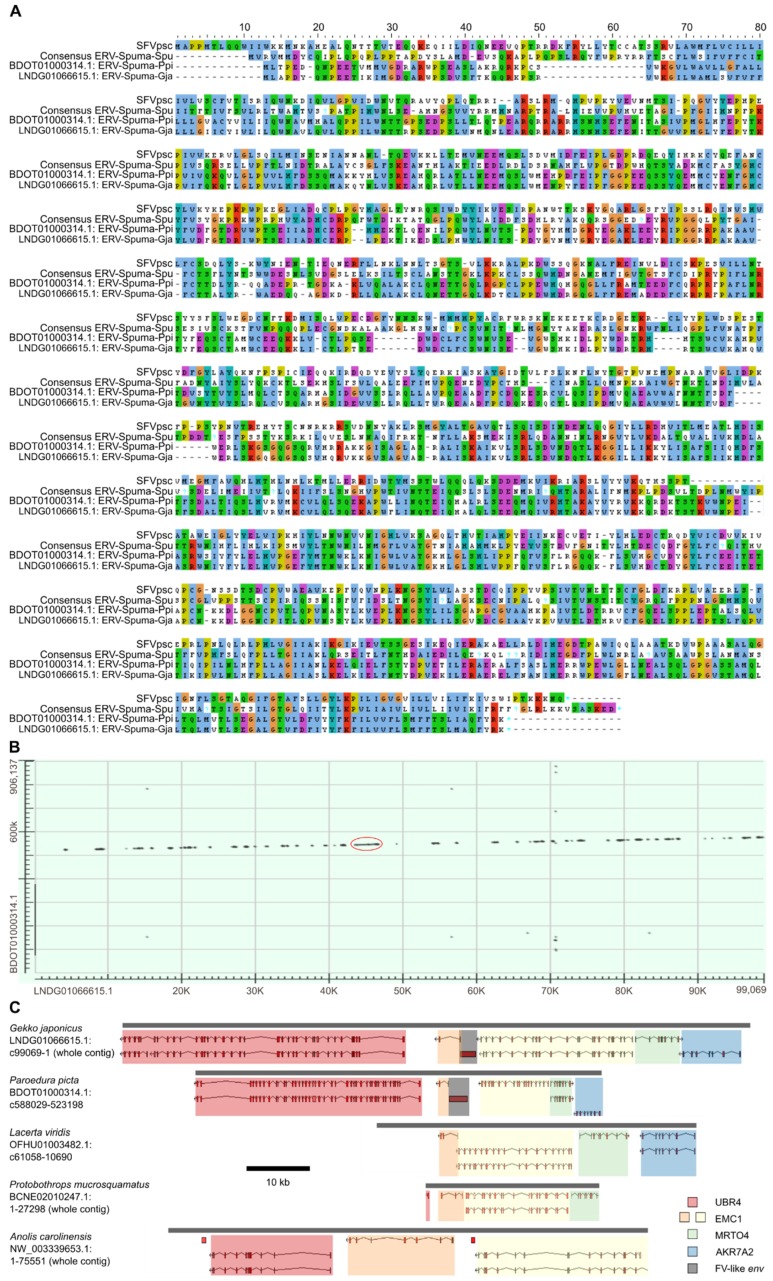
FV-like *env* sequences in panther gecko (*Paroedura picta*, BDOT01000314.1) and Schlegel’s Japanese gecko (*Gekko japonicus*, LNDG01066615.1). (**A**) Alignment of Env protein sequences of prototype FV (SFVpsc), consensus ERV-Spuma-Spu, and the two gecko FV-like endogenous viral elements, BDOT01000314.1: ERV-Spuma-Ppi, and LNDG01066615.1: ERV-Spuma-Gja. (**B**) BLASTn dot matrix between LNDG01066615.1 and BDOT01000314.1. The red circle indicates the location of the FV-like *env* sequences. (**C**) Homologous regions in three other reptiles, namely European green lizard (*Lacerta viridis*, OFHU01003482.1), brown spotted pitviper (*Protobothrops mucrosquamatus*, BCNE02010247.1), and green anole (*Anolis carolinensis*, NW_003339653.1). Genes were predicted by AUGUSTUS [9]. Four eukaryotic genes are shown on the diagram from left to right: ubiquitin protein ligase E3 component n-recognin 4 (UBR4, red), ER membrane protein complex subunit 1 (EMC1, orange and yellow), MRT4 homolog, ribosome maturation factor (MRTO4, green), and aldo-keto reductase family 7 member A2 (AKR7A2, blue). Gene homology at the protein level was examined by using BLASTp. FV-like env genes are highlighted in grey. Grey bars represent the contigs, and the scale bar (black) represents a length of 10 kb.

The contigs containing ERV-Spuma-Ppi and ERV-Spuma-Gja were co-linear, suggesting that they may be orthologs. Genes surrounding the EFVs were determined and compared to confirm orthology. While LNDG01066615.1 was annotated with genes, BDOT01000314.1 was not. We thus used the gene prediction program AUGUSTUS [9] to annotate the contig. Homologous regions in other reptiles, including the European green lizard (*Lacerta viridis*, OFHU01003482.1), the brown spotted pitviper (*Protobothrops mucrosquamatus*, BCNE02010247.1), and the green anole (*Anolis carolinensis*, NW_003339653.1), were identified based on the genes found on LNDG01066615.1 and BDOT01000314.1 by using tBLASTn. AUGUSTUS [9] was used to annotate the genes on these contigs when gene annotations were not available. The results are shown in Figure 2.

To determine the type of selection that ERV-Spuma-Ppi and ERV-Spuma-Gja were under, their nucleotide sequences were aligned, and a dN/dS ratio was computed by using CodeML implemented in PAML 4.9e [10]. The run mode was set to the “pairwise” mode (runmode = −2). No clock was assumed (clock = 0: no clock) with all sites assumed to have evolved under the same rate (fixed α = 1 and α = 0), and the equilibrium codon frequencies were assumed to be equal to those that were observed (CodonFreq = 3: code table; estFreq = 0: use observed freqs). The universal genetic code (icode = 0: universal) was used to determine the number of synonymous and non-synonymous sites and changes. The basic model of selection (model = 0: one dN/dS ratio; NSsites = 0: one ω) was used to compute an overall dN/dS ratio for the entire *env* like gene. 

To investigate the possibility that ERV-Spuma-Ppi and ERV-Spuma-Gja might be transcriptionally active, we used them to query the Reptilian Transcriptomes v2.0 Database [11], using both BLASTn and tBLASTn searches. One very significant hit was returned from the transcriptomic sequence database of common leopard Gecko (*Eublepharis macularius*), named “EMA_Contig_Illumina_8645”. It was a consensus contig that was estimated from 499 raw transcript reads. 

### 2.4. Recombination Analyses

A concatenated alignment of *pol*-*env* nucleotide sequences of mammalian, tuatara, amphibian, and lobe-finned fish FVs/EFVs was prepared for recombination analyses. Their *gag* sequences were not included, as they could not be aligned among these viruses. To obtain ERV-Spuma-Spu’s *pol* and *env* sequences, we used BLASTn to query the 165 ERV-Spuma-Spu elements with the consensus ERV-Spuma-Spu’s *pol* and *env* sequences with an E-value cut off of 1 × 10^−6^. Hits that were found in the same ERV-Spuma-Spu element and that were in the same orientation were concatenated according to their hit locations to obtain contiguous viral sequences without transposable elements. We only included those with both *pol* and *env* gene coverage of ≥ 80% in the alignment. The final alignment contained 45 sequences, 27 of which were ERV-Spuma-Spu elements and were 4695 nucleotides (nt) long (*pol*: 2685 nt; *env*: 2010 nt) (Data S4).

Potential recombination events were detected using 7 programs: RDP, GENECONV, Chimaera, MaxChi, BootScan, SiScan, and 3Seq, implemented in Recombination Detection Program 4 [12] with their default settings. Only those detected with >4 programs were considered significant.

### 2.5. Phylogenetic Analyses

**Figure 3:**
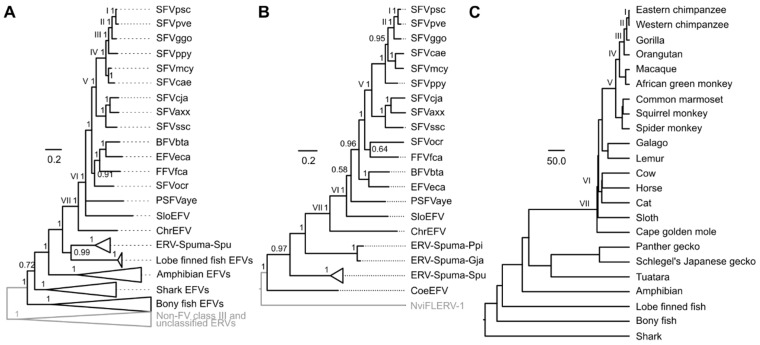
Foamy virus Pol, Env, and host phylogenies. Bayesian Pol (**A**) and Env (**B**) phylogenies were estimated by using MrBayes 3.2.6 [13], and their scale bars are in the units of amino acid substitutions per site. Both Pol and Env trees were rooted by the mid-point rooting method, and the determined outgroups are shown in grey. Arabic numerals on nodes are Bayesian posterior probability clade support values. The topologies of the Pol and the Env trees were compared to that of the host phylogeny (**C**) to identify virus–host co-speciation events labeled with Roman numerals. Nodes on different phylogenies that are labeled with the same Roman numeral are those corresponding to the same co-speciation event. The timescale of the identified co-speciation nodes, directly inferred from their hosts (Table S5), was used to calibrate the timescales of other nodes. The host tree topology was estimated elsewhere [26], and its scale bar is in units of millions of years. The virus–host association can be found in Table S6. SFV: simian foamy virus; psc, *Pan troglodytes schweinfurthii* chimpanzee; pve, *Pan troglodytes verus* chimpanzee; ggo, *Gorilla gorilla* gorilla; *ppy*, *Pongo pygmaeus* orangutan; mcy, *Macaca cyclopis* macaque; cae, *Chlorocebus aethiops* Grivet; cja, *Callithrix jacchus* marmoset; axx, Ateles spider monkey; scc, *Saimiri sciureus* squirrel monkey; ocr, *Otolemur crassicaudatus* brown greater galago; BFVbta, bovine foamy virus Bos taurus; EFVeca, equine foamy virus Equus caballus; FFVfca, feline foamy virus Felis catus; PSFVaye, prosimian foamy virus aye-aye; EFV, endogenous foamy virus; SloEFV, sloth EFV; ChrEFV, Cape golden mole EFV; CoeEFV, Coelacanth EFV; NviFLERV-1, Notophthalmus viridescens foamy virus-like endogenous retrovirus - 1.

Recombination analyses suggested that FVs’ *pol* and *env* genes might have different evolutionary histories (see Results). We thus estimated Pol and Env protein phylogenies separately under the Bayesian phylogenetic framework by using MrBayes 3.2.6 [13] to better understand how they evolved (Figure 3). The Env protein alignment was derived from the *env* nucleotide alignment used in the recombination analyses with the addition of sequences from the two gecko EFVs (Data S5). For the Pol protein alignment, sequences from fish EFVs and non-FV class III ERVs were included as an outgroup (Data S6). This alignment (containing only the reverse transcriptase and integrase coding domain portions) was based on the one we used in our previous study, allowing the results to be compared. The best-fit amino acid substitution models were determined to be JTT+I+Γ(4)+F for the Env alignment and JTT+Γ(4)+F for the Pol alignment by ProtTest 3.4.2 [14] under the sample size corrected Akaike information criterion. Two Markov chain Monte Carlo chains were run for 10,000,000 steps with a sampling frequency of 1 per 1000 steps. The metropolis coupling algorithm (3 hot chains and 1 cold chain) was used to improve the sampling. The first 25% of sampled parameter values were discarded as burn-in. Potential scale reduction factors of all parameters were ~1.000 in both analyses, indicating that they were all well sampled from their posterior distributions and had converged.

### 2.6. Evolutionary Timescale Inference

Many studies have shown that mammalian FVs have a long-term co-speciation history with their hosts throughout the entire evolution of the eutheria [2,15,16,17]. This extraordinarily evolutionary feature has led to the observation that the relationship between the virus total per-lineage substitution numbers (s) and the evolutionary timescales (t) can be approximated very well by a power-law function: log(t)=αlog(s)+β [18]. We used this relationship, the so-called time-dependent rate phenomenon (TDRP) model, to estimate evolutionary timescales of reptile EFVs.

For each of the trees in the posterior distribution obtained from the Bayesian phylogenetic analyses, we traced the simian foamy virus Pan troglodytes verus (SFVpve) backwards in time to various virus–host co-speciation nodes to obtain various s estimates, of which the corresponding t estimates could be inferred directly under the co-speciation assumption (Table S5). Based on virus–host tree topology comparison, seven co-speciation nodes could be inferred in the Pol phylogeny (Figure 3A, labeled with black Roman numerals), and five were inferred in the Env tree (Figure 3B, labeled with black Roman numerals) down the SFVpve lineage. These corresponding s and t estimates were used to calibrate the TDRP model. The model fitting was performed by using the *lm* function implemented in R 3.1.2 [19], and the t and the s estimates were log-transformed (base 10) prior to the linear model fitting. The model was then extrapolated to estimate the timescales of other nodes from their s estimates.

## 3. Results

### 3.1. Characterisation of ERV-Spuma-Spu

By using a series of BLAST searches (see Materials and Methods for details), we identified 165 FV-like endogenous viral elements (EVEs) in the genome of tuatara (*Sphenodon punctatus*; accession number: QEPC01000000). All of these EVEs showed the greatest similarity to modern FV Pol protein sequences (and not to those of other retroviruses) and harbored FV-like *env* sequences (Tables S1–S3). We observed that most of these EVEs contained transposable elements, interrupting their protein coding regions, indicating that they are genuine non-functional and old endogenous viruses and not contamination of extant virus sequences in the sequence of the tuatara genome. Due to the poor quality of the tuatara genome at the time of analysis, many EVE sequences contained large strings of “N”, representing undetermined nucleotide sequences. We treated them as gaps in the downstream analyses in this study.

A consensus sequence of these FV-like EVEs (Figure 1A) was reconstructed for genome annotation (see Materials and Methods for details). Twenty out of the 165 elements that showed the greatest similarity to the concatenated CoeEFV’s Pol and Env protein sequence were aligned and curated and subsequently used to reconstruct a consensus sequence. The consensus sequence of the virus body was inferred separately from the long terminal repeat (LTR) portion (Data S1), and the consensus LTR sequence was inferred from both 5’- and 3’-LTR sequences (Data S2). An average pairwise distance between these 20 elements was estimated to be only 0.118 substitutions per site (body portion), and they could be aligned with high confidence, indicating that they are of the same virus lineage. See Figure S1 and Data S3 for the full consensus sequence. Sequence comparison revealed that this virus is highly similar to the ERV-Spuma-Spu previously reported in [8]. They exhibit 97.17% nucleotide percentage identity and 97% coverage (with the one in [8] being 3% shorter), suggesting they are the same virus. The slight differences are likely due to the different methods we used to reconstruct the sequences.

In summary, the consensus sequence was 10,044 nucleotides (nt) long. The LTRs were 935 nt in length (5’-LTR: nt 1–935, 3’-LTR: nt 9110–10,044), which were longer than those of other retroviruses such as alpharetroviruses (~350 nt), gammaretroviruses (~600 nt), deltaretroviruses (~550–750 nt), and lentiviruses (~600 nt), but were typical for FVs (~950–1700 nt) [20]. We noted that our LTRs were longer than those reported in [8] (694 nt); a sequence comparison showed that the consensus sequence reported in [8] was missing ~240 nt corresponding to the beginning (i.e., the 5’ end) of the LTRs (Data S2). A lysine tRNA utilizing primer binding site (PBS) was identified downstream of the 5′-LTR (TGGCGCCCAAYGTGGGGCTCGA, nt 938–959), which is typical of mammalian FVs [21,22]. The *gag* gene was predicted to be 1308 nt long (nt 1085–2392) to generate a 435 amino acid (aa) protein. This was markedly shorter than those of mammalian FVs (~550–650 aa). The *pol* and *env* genes were determined to be 3540 nt (nt: 2487–6026; 1179 aa) and 3003 nt (nt: 5956–8958; 1000 aa) long, respectively, which are typical lengths of mammalian FV *pol* and *env* genes. Results from reciprocal BLAST analyses revealed that all three protein products were most similar to those of mammalian FVs (Table 1), consistent with previously reported results [8]. Phylogenetic analysis also showed that these EVEs clustered with other FVs and EFVs (see below), supporting that this consensus sequence is indeed derived from EFV elements.

One hypothetical open reading frame (ORF) was identified as an accessory gene in the previous study [8]. This ORF could be mapped to nt 9114–9498 in our consensus sequence, corresponding to the LTR portion (Data S2). In our consensus sequence, the identified ORF appeared to be broken into two separate ORFs of different frames (nt 9114–9305, frame +3 and nt 9343–9498, frame +1; Figure 1A). The crucial difference was that the Wei et al. sequence missed a nucleotide, causing the two ORFs to merge as one (Data S2; 10049th column in the alignment; 196th nt in our consensus LTR sequence). Indeed, we failed to locate any accessory genes between the *env* gene and the 3’-LTR, typical of a mammalian FV (Figure 1A). In addition, as previously noted, the hypothetical accessory protein did not exhibit significant similarity to any known FV proteins [8] or indeed any molecular sequences in the National Center for Biotechnology Information (NCBI) non-redundant (nr) nucleotide collection database. Furthermore, we could not identify an internal promoter towards the 3’ end of the *env* gene (Figure 1B) required for efficient accessory gene expression [23,24]. All these results suggest that ERV-Spuma-Spu may actually lack accessory genes and that the previously identified accessory gene was an artifact.

### 3.2. The Discovery and Characterisation of Gecko EFVs

In addition to ERV-Spuma-Spu elements, we were able to recover two FV-like elements from two gecko genomes, namely panther gecko (*Paroedura picta*; accession number: BDOT01000000) and Schlegel’s Japanese gecko (*Gekko japonicus*; accession number: LNDG01000000). They were found on contig BDOT01000314.1 (nt: c548,029–545,198; 943 aa) and contig LNDG01066615.1 (nt: c46,188–43,360; 942 aa), respectively. Both elements consisted solely of a full-length FV-like *env* gene, and no other FV-like elements could be identified. Interestingly, neither of them contained any in-frame stop codons or transposable elements. Reciprocal BLAST analyses showed that both elements were most similar to modern mammalian FVs (Table S4). Consistent with this finding, phylogenetic analysis revealed that they clustered with other FVs and EFVs (see below). Combined, these results strongly suggest that they are EFVs. We designated the two elements ERV-Spuma-Ppi and ERV-Spuma-Gja, respectively.

We determined that BDOT01000314.1 and LNDG01066615.1 were co-linear, suggesting that ERV-Spuma-Ppi and ERV-Spuma-Gja might be orthologous. ERV-Spuma-Gja was found in the intronic region of the predicted endoplasmic reticulum membrane protein complex subunit 1 gene (EMC1: XM_015412627.1) between exon 21 and 22 in the antisense orientation. Further examination revealed three other genes in the same vicinity of ERV-Spuma-Gja, namely ubiquitin protein ligase E3 component n-recognin 4 gene (UBR4: XM_015412557.1), MRT4 homolog, ribosome maturation factor gene (MRTO4: XM_015412607.1), and aldo-keto reductase family 7 member A2 gene (AKR7A2: XM_015412595-6.1). At the time of analysis, the contig BDOT01000314.1 (of the panther gecko genome) was not annotated with genes; nevertheless, we were able to confirm that ERV-Spuma-Ppi had the same genomic location as ERV-Spuma-Gja and was surrounded by the same set of genes in the same order, confirming that they are orthologs. Moreover, we were able to identify corresponding homologous genomic regions in the genomes of European green lizard (*Lacerta viridis*, OFHU01003482.1), brown spotted pitviper (*Protobothrops mucrosquamatus*, BCNE02010247.1), and green anole (*Anolis carolinensis*, NW_003339653.1) based on the presence of these genes. However, none of these reptile genomes had homologs of ERV-Spuma-Ppi and ERV-Spuma-Gja. The results are shown in Figure 2. We thus could infer that ERV-Spuma-Ppi and ERV-Spuma-Gja are at least 96 (83–98) million years (myr) old based on the speciation date of panther gecko and Schlegel’s Japanese gecko [25]. Furthermore, a pairwise nucleotide sequence comparison of ERV-Spuma-Ppi and ERV-Spuma-Gja estimated its dN/dS ratio to be 0.14 (0.08–0.54), strongly suggesting that they were under strong purifying selection pressure.

Remarkably, when we queried the Reptilian Transcriptomes V2.0 Database [11] with ERV-Spuma-Ppi and ERV-Spuma-Gja, we recovered a transcript consensus contig from the transcriptome of common leopard gecko (*Eublepharis macularius*), which is closely related to panther gecko and Schlegel’s Japanese gecko. The sequence exhibited 82.2 and 93.0 aa percentage identity (E-value = 4 × 10^−66^ and 2 × 10^−74^) and 81.6 and 86.94 nt percentage identity (E-value = 1 × 10^−93^ and 4 × 10^−118^) to ERV-Spuma-Ppi and ERV-Spuma-Gja, respectively. The contig was 626 bases long and constructed from 499 raw reads. We could not check for the homolog of ERV-Spuma-Ppi and ERV-Spuma-Gja in the leopard gecko genome, as it is currently not available. However, this finding suggests that ERV-Spuma-Ppi and ERV-Spuma-Gja might be transcriptionally active.

### 3.3. Recombination Analyses

A concatenated nucleotide alignment of *pol* and *env* sequences of ERV-Spuma-Spu elements together with those of mammalian, amphibian, and lobe-finned fish FVs and EFVs was checked for potential recombination events by Recombination Detection Program 4 (RDP4) [12]; *gag* sequences were not included, as they could not be aligned among these viruses. We found that, out of 165 ERV-Spuma-Spu elements, 138 of them (83.6%) had coverage of either *pol* or *env* genes of < 80% (see Methods and Materials for details), likely due to the poor genome quality at the time of analysis. We thus decided to exclude them from any further downstream analyses. The alignment contained 45 sequences, 27 of which were those of ERV-Spuma-Spu elements, and was 4695 nt (*pol*: 2685 nt; *env*: 2010 nt) long after curation (Data S4).

The analysis first identified two ERV-Spuma-Spu elements as recombinants (QEPC01002018.1: 1489335–1511987, and QEPC01002018.1: 1489335–1511987), harboring an integrase coding domain of unknown origin at the same genomic locations (position in the alignment: nt 2038–2596; Figure S2). We removed the recombinant regions (nt 2152–2432 after manual inspection) and performed the analysis again to further examine for other potential recombination events.

The results from the second round of analysis suggested that the *pol* and the *env* genes have different evolutionary histories. Based on the default dendrogram outputs from RDP4 [12] estimated using the unweighted pair group method with arithmetic mean, we found that, while ERV-Spuma-Spus’ *pol* genes are more closely related to those of CoeEFV, their *env* genes are more similar to those of mammalian FVs (Figure S3). No recombination could be detected in either of the individual *pol* and *env* nucleotide alignments after that. 

### 4.4. Phylogenetic Analyses and Evolutionary Timescale Estimation

To better understand how the *pol* and *env* genes evolved, their evolutionary histories were estimated from their corresponding protein alignments by using a Bayesian phylogenetic method (Figure 3). The Env protein alignment was derived from the *env* nucleotide alignment used in the recombination analyses with the addition of the two gecko EFVs to investigate how they relate to other FVs (Data S5). For the Pol protein alignment, we included sequences from fish EFVs and non-FV class III ERVs as an outgroup. This Pol alignment (Data S6) was based on the one we used previously in the study reporting the discovery of amphibian and fish EFVs [4], allowing the results to be compared.

Overall, the well-established broad co-speciation pattern between mammalian FVs and their hosts could be recovered from both the Pol and the Env phylogenies (Figure 3), and the topology of the Pol tree was comparable to that previously published in [4]. Our analyses suggested that ERV-Spuma-Spus’ Pol is sister to that of CoeEFV (Bayesian posterior probability clade support = 0.99), while their Env is more closely related to those of mammalian FVs (Bayesian posterior probability clade support = 0.97). These results were consistent with those obtained from the recombination analysis (Figure S3). In addition, we found that the Env proteins of ERV-Spuma-Spu elements did not cluster with gecko EFVs; they instead formed two separate lineages, with gecko EFVs being closer to mammalian FVs (Bayesian posterior probability clade support = 1.00). In addition, we noted that, while our Pol protein analysis strongly supports the sister taxon relationship between ERV-Spuma-Spu and CoeEFV, the previous Pol protein analysis showed that ERV-Spuma-Spu is a sister taxon of mammalian FVs, inferred under the maximum likelihood framework with 89% bootstrap support [8]. This could be due to the differences in the methods (Bayesian vs. maximum likelihood) and/or the alignments used (reversed transcriptase + RNase-H + integrase domain vs. reversed transcriptase + RNase-H).

Evolutionary timescales of reptile EFVs were estimated using the time dependent rate phenomenon (TDRP) model [18,27]. The TDRP model is a model that describes the relationship between total per lineage substitutions (s estimates) and their associated evolutionary timescales (t estimates) using a power law function ($t=\alpha s^{\beta }$), and this relationship can be used to estimate a t value for an arbitrary node given its s (node height in the units of substitutions per site) [18,27]. We traced simian foamy virus Pan troglodytes verus (SFVpve) down the trees to obtain total per lineage substitutions across various timescales for the TDRP model estimation. Based on virus–host tree topology comparison, we inferred seven virus–host co-speciation events in the Pol phylogeny (Figure 3A, labeled with Roman numerals) and five in the Env tree (Figure 3B, labeled with Roman numerals) that lie along the SFVpve lineage, and the timescales of these nodes were inferred directly from those of their hosts (Table S5). Two TDRP models were estimated based on the t and the s estimates of these identified co-speciation nodes, one for the Pol protein [α = 364.08 (242.33–533.43), β = 1.59 (1.34–1.86), Adjusted R^2^ = 0.95 (0.90–0.99)], which was comparable to the one previously reported {α = 407.89 (264.32–583.97), β = 1.63 (1.38–1.90), Adjusted *R*^2^ = 0.95 (0.91–0.99) [4]}, and the other for the Env protein [α = 124.18 (100.42–152.36), β = 1.41 (1.21–1.60), Adjusted R^2^ = 0.96 (0.91–0.99)]. They were then used to extrapolate in order to calculate the timescales of other nodes based on their s estimates.

Analyses of the Pol protein sequences suggested that the ERV-Spuma-Spu lineage diverged 232.50 (173.70–303.33) myr ago (mya), which was comparable to that estimated based on the Env protein sequences, which was 257.15 (202.49–324.05) mya. These age estimates were, however, significantly lower than those of their hosts, which were estimated to be 324.7 (318–331.4) mya [28]. The age of the gecko EFV lineage was estimated to be 208.54 (171.59–250.91) myr old based on phylogenetic analysis of the Env protein sequences. This was consistent with their minimum age estimate of ~96 myr old. In addition, based on the Pol phylogeny, we also estimated the age of the amphibian EFV lineage and the entire clade of vertebrate FVs/EFVs to be 326.40 (229.14–448.65) myr old and 479.08 (298.31–718.86) myr old, respectively. These estimates were comparable to those previously reported {amphibian FVs: 348 (251–478) myr old and vertebrate FVs: 455 (304–684) myr old [4]} and those of their hosts (amphibians: ~335 myr old [29], and vertebrates: ~465 myr old [29]). Our results are thus consistent with those previously reported and support the long-term co-evolution between FVs and their hosts since the origin of vertebrates.

## 4. Discussion

This study reports two novel reptile EFVs that reside in the genomes of panther gecko (ERV-Spuma-Ppi) and Schlegel’s Japanese gecko (ERV-Spuma-Gja) and further characterizes ERV-Spuma-Spu [8]. Together with ERV-Spuma-Spu [8], we analyzed the evolutionary history of reptilian FVs in detail, filling in the gap in our knowledge of the deep history of FV-host co-evolution.

ERV-Spuma-Ppi and ERV-Spuma-Gja are not full length ERVs, comprising only a full-length FV-like *env* gene, and are present in only a single copy in each genome. We showed that they are orthologous, being present in the same host genomic location in both species. Based on the speciation date of their gecko hosts, we inferred that these EFVs are at least 96 (83–98) myr old [25], making them the oldest EFVs ever discovered to date. The two gecko EFVs are located in the intronic region of the EMC1 gene in an antisense orientation typical of an old fixed intronic ERV; antisense integrations are favored because they are likely minimally disruptive to the host’s gene transcription processes [30]. Remarkably, they do not contain any in-frame stop codons or transposable elements despite being almost 100 myr old and have been under strong purifying selection with a dN/dS estimate of 0.14 (0.08–0.54). Furthermore, by searching the transcriptomic sequence database of the common leopard gecko in the Reptilian Transcriptomes v2.0 Database [11], we discovered a transcript consensus sequence constructed from 499 reads that is highly similar to ERV-Spuma-Ppi and ERV-Spuma-Gja, exhibiting more than 80 percent identity both at the protein and the nucleotide levels. Combined, these findings suggest that they might be transcriptionally active and have been maintained for potential cellular functions, making them the first ever known co-opted EFVs. We, however, noted that the transcript we retrieved was distinct from the two gecko EFVs obtained from a different host species and mapped to only a small portion of the 3’ end of the *env* genes. Additional analyses of transcriptomic data obtained directly from *Gekko japonicas* and *Paroedura picta* are thus required to confirm that the two gecko EFVs are transcriptionally active.

Retroviral *env* genes are known to have been co-opted many times by various vertebrate hosts for a wide range of functions. The most well-known one is perhaps the *syncytin* genes, which was captured for a function in placental formation numerous times by various mammals (see [31,32] for reviews). A recent study identified (for the first time) a functionally active *syncytin* gene outside mammals, namely *syncytin-Mab1*, in a lizard *Mabuya* [33,34]. The reptilian *syncytin* gene was identified as a gammaretrovirus *env* gene, however, which belongs to a different group to our two gecko EFVs. Furthermore, *Mabuya* is a viviparous placental lizard, while geckos are not. Thus, the functions of ERV-Spuma-Ppi and ERV-Spuma-Gja might differ from that of *syncytin-Mab1.*

The fact that the *env* reading frames are still intact and under purifying selection despite their old age suggests that ERV-Spuma-Ppi and ERV-Spuma-Gja are functional at the protein level. BLASTp analyses against the NCBI nr protein database failed to identify cellular proteins that are related to ERV-Spuma-Ppi and ERV-Spuma-Gja. Therefore, their functions still remain elusive. Nonetheless, the two gecko EFVs are inserted in the EMC1 gene, which intriguingly shows greatest expression level in placenta tissues in human [35]. One could thus imagine that the two genetic elements might be co-expressed and are in turn functionally associated, as has been repeatedly shown in several organisms [36,37,38]. Although geckos lay eggs and do not possess true placenta tissues such as *Mabuya* lizards, the physical association of EMC1 with co-opted retroviral *env* sequences suggests their possible involvement in the reproductive system of geckos, analogous to the *syncytin* gene in mammals [31,32] and viviparous placental lizards [33,34]. Furthermore, the EMC1 protein influences virus cross-membrane transportation and infectivity via direct physical contact with viral particles [39]. The two gecko EFVs might thus play an active role in the host immune systems as well, if their functions are indeed associated with those of EMC1. Indeed, studies have shown that retroviral *env* genes can be co-opted and exapted for host anti-viral defense, acting as restriction factors against related retroviruses [40,41]. Our finding warrants further functional investigation to confirm the potential involvement of ERV-Spuma-Ppi and ERV-Spuma-Gja in gecko reproductive and/or immune systems.

We were able to identify 165 ERV-Spuma-Spu elements in the tuatara genome. Examination of their consensus sequence revealed that ERV-Spuma-Spu only possessed the three main retroviral core genes, namely *gag*, *pol*, and *env*, flanked by two LTRs. A previous study identified one short hypothetical open reading frame (192 nt) as an accessory gene [8]; however, we found that the sequence was actually part of the 3’-LTR. On the other hand, we could not identify any accessory genes located between the *env* gene and the 3’ LTR, which is typical for a mammalian FV [4,6,42]. The authors of the previous study also noted that the identified accessory gene did not exhibit similarity to any known foamy accessory genes. Further examination revealed that the hypothetical protein was not intact and did not exhibit significant similarity to any known molecular sequences in the NCBI nr database. In addition, we could not identify a potential internal promoter towards the 3’ end of the *env* gene. It is thus possible that the previously identified accessory gene in ERV-Spuma-Spu might have been an artifact. At face value, the observed lack of accessory genes is suggestive of ancient gene losses in ancestral exogenous reptile FVs. Alternatively, it could be that the ancestral exogenous tuatara FVs did possess accessory genes but lost them after becoming endogenous. This observation is also consistent with multiple acquisitions of accessory genes in other FVs at the same genomic location, which is perhaps less parsimonious but nevertheless possible. Discovery of other reptilian FVs or EFVs will help elucidate this issue.

Furthermore, we found that the ERV-Spuma-Spu *gag* gene was markedly shorter than those of typical simian FVs. Protein sequence comparison showed that the putative ERV-Spuma-Spu Gag protein had a full matrix domain essential for Gag–Gag interaction [43,44], Gag–Env interaction [44], and Gag–microtubular network interaction [45]. The conserved central region, which is evolutionarily related to orthoretroviral capsid proteins [46], could also be found. The regions between the matrix and the capsid domain (aa ~180–~300 of the SFVpsc Gag protein) were missing, however, containing the late domain (P_284_SAP domain), which mediates viral particle release [47]. Nevertheless, this region was not highly conserved; indeed, non-primate FVs including bovine, equine, and feline FVs also lack this region and the late domain [48]. 

**Figure 4:**
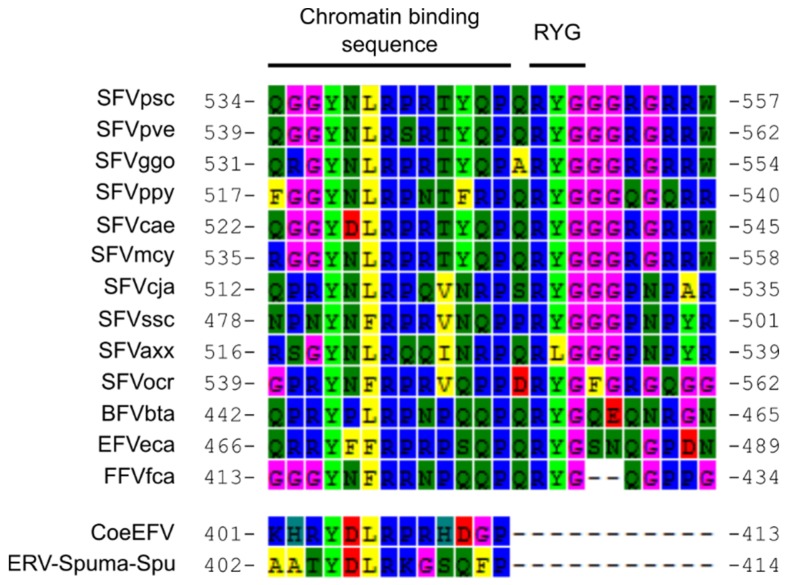
An alignment of chromatin-binding sequences (CBS) and surrounding regions. CBSs of CoeEFV and ERV-Spuma-Spu are intact. The conserved tyrosine (Y; 4^th^ column) and arginine (R; 7^th^ column) residues in the CBS, which are essential for Gag mitotic chromosome binding and nuclear accumulation of Gag and genomic DNA [49,54,55], could be found in all viruses. The arginine-tyrosine-glycine (RYG) residues, which are also important for nuclear accumulation of FV Gag and DNA [55], were absent from CoeEFV and ERV-Spuma-Spu but could be found in all mammalian FVs.

Moreover, a region homologous to the C-terminus of the nucleocapsid domain (corresponding to aa 549–648 in the SFVpsc Gag protein) containing part of the glycine/arginine rich box (GR) II and the entire GR-III was also missing from the putative ERV-Spuma-Spu Gag protein. GR-I, a nucleolar localization signal [49] that mediates nucleic acid binding [50] and is important for Pol packaging [51] and particle formation [52], could still be found. Sequence examination revealed that the chromatin-binding sequence (CBS) in GR-II (aa 534–546 in the SFVpsc Gag protein [53]) required for a direct physical contact between FV Gag protein and host nucleosomes [54] was still intact (Figure 4). The conserved tyrosine and arginine residues in the CBS (Y405 and R408) could also be found (Figure 4), and are essential for Gag chromosome binding and nuclear accumulation of Gag and genomic DNA [49,54,55]. The arginine-tyrosine-glycine (RYG) residues following the CBS (Figure 4) are crucial for nuclear accumulation of FV Gag and DNA, and, perhaps most importantly, DNA integration [55]. Mutagenesis of these residues causes significant reduction in all three activities, even if the CBS is complete [55]. Intriguingly, the RYG domain is absent from both ERV-Spuma-Spu and CoeEFV (Figure 4), which are incidentally the only two EFVs known to be present in high copy numbers in the host genomes [5,8]. In addition, the missing GR-III box was shown to be a nucleolar localization signal similar to GR-I [49]. Studies have shown that the deletion of GR-III only marginally affects viral budding [52,56], intracellular localization [52,57], reverse transcription [52], RNA packaging/binding [50,52,56,58], and particle morphology [52] but significantly reduces DNA packaging [52] and thus infectivity [52,56]. The lack of GR-III box and the RYG residues might help with the virus retrotransposition process by allowing their DNA to accumulate in the host cell and subsequently re-integrate into the host chromosomes in a steady and non-aggressive manner. As previously reported [8], a number of ERV-Spuma-Spu elements of different ages with paired-LTRs could be recovered. This means that at least some of the ERV-Spuma-Spu elements originated from the re-integration process and not via host genomic copying or LINE-mediated retrotransposition of viral mRNA, supporting our hypothesis. These observations might underlie the high copy number of ERV-Spuma-Spu and CoeEFV elements found in the tuatara [8] and the coelacanth genomes [5], respectively.

Based on their observation that ERV-Spuma-Spu is more closely related to mammalian FVs than CoeEFV, Wei et al. proposed an ancient co-speciation of ERV-Spuma-Spu and mammalian FVs dating back more than 320 million years ago [8]. This study, on the other hand, estimated the dates directly based on molecular analyses of Pol and Env protein sequences and the more well-established history of mammalian FV-host co-speciation. Our evolutionary analyses of Pol and Env proteins showed that the ERV-Spuma-Spu lineage is only 232.50 (173.70–303.33) and 257.15 (202.49–324.05) myr old, respectively, comparable to one another in age. These estimates are much lower than those of their hosts, ~324.7 myr old [28]. This finding rejects the ancient co-speciation hypothesis previously proposed and instead suggests that the ancestral virus that gave rise to ERV-Spuma-Spu elements arose from (potentially a series of) cross species transmission(s) from an unknown, non-reptilian host. This result also highlights the pitfall of using tree topologies alone to infer a virus–host co-speciation history, especially when there are only a few lineages in the investigation.

Our phylogenetic analyses of Env proteins revealed that, while ERV-Spuma-Ppi and ERV-Spuma-Gja form a clade, they do not form a monophyletic clade with ERV-Spuma-Spu elements, with the two gecko EFVs being closer to mammalian FVs than ERV-Spuma-Spu (Bayesian posterior probability clade support = 1.00). We estimated the age of the gecko EFV lineage to be 208.54 (171.59–250.91) myr old. Again, this low age estimate is suggestive of a cross-species transmission origin for the gecko EFVs’ ancestor, one that is broadly contemporary but independent from the transmission that gave rise to the ancestor of ERV-Spuma-Spu elements.

The phylogenetic placement of CoeEFV with respect to that of the ERV-Spuma-Spu lineage also suggests an evolutionary history of cross-species transmission. While the Pol protein of CoeEFV exhibits a sister relationship with that of ERV-Spuma-Spu viruses (Figure 3A), its Env protein does not, instead being basal to the clade of mammalian and reptile FVs (Figure 3B). Since CoeEFV forms a clade with ERV-Spuma-Spu viruses in the Pol phylogeny, their branching dates from mammalian FVs are hence the same, estimated to be 232.50 (173.70–303.33) mya. This is indeed comparable to the previously reported estimate of 262.76 (195.00–342.08) mya [4] and further supports the complex evolutionary history of FVs that might have transmitted several times between terrestrial and aquatic animals in the distant past [4].

On the other hand, we estimated CoeEFV’s Env protein (and thus its *env* gene) to share a most recent common ancestor with that of mammalian FVs 332.68 (236.13–451.42) mya. This age estimate is drastically older than that of the *pol* gene, suggesting that CoeEFV’s *pol* and *env* genes might have different evolutionary histories. We note however that this age estimate is conditioned on how the Env tree is rooted. In this study, we chose the mid-point rooting method, which placed NviFLERV-1 (the amphibian EFV) as the most basal lineage in the Env tree (Figure 3B), consistent with the topology of the Pol tree (Figure 3A). However, if the *pol* and the *env* genes can have different evolutionary histories, then there would be no intrinsic reasons for the Pol and the Env tree topologies to closely resemble each other. Another possibility is to subjectively place CoeEFV as the most basal linage in the Env tree, in which case the estimated date would be the divergence date of NviFLERV-1 instead, which in turn would make it a lower bound estimate for the branching date of CoeEFV’s *env* gene. This nonetheless still supports the hypothesis that CoeEFV’s *pol* and *env* genes have different evolutionary histories. 

This finding mirrors observations from primate [59] and feline FVs [60,61]. Studies at the population level identified the surface domain of their *env* genes to have evolutionary histories that are strikingly different from the rest of the *env* gene and the *pol* gene [59,60,61,62], segregating into two variants that co-circulate in the same host populations while other genomic regions are not phylogenetically distinguishable. This domain carries the receptor binding domain and is targeted by neutralizing antibodies [60,63], which may help explain its greater diversity. Our analyses could not detect this evolutionary feature, since our dataset comprises only one sequence from each FV species. Nonetheless, it is remarkable that a similar evolutionary pattern could still be observed at the species level focusing on different timescales. Our results thus further support the modular nature of FV genomes and that this might be a widespread evolutionary feature of FVs.

Our analyses reveal a complex evolutionary history and ancient transmission routes of ancient FVs, likely involving host switches across the boundary between water and land, as well as the modular nature of their genomes. Our work also highlights the importance and the value of recombination analysis and temporal information in evolutionary inference as well as the pitfalls of tree-topology based virus–host co-speciation analysis. Discovery of additional EFVs will undoubtedly further our understanding and improve our knowledge of the complex and rich natural history of FVs.

## Figures and Tables

**Table 1:** Reciprocal BLASTp analyses of ERV-Spuma-Spu against the National Center for Biotechnology Information (NCBI) non-redundant retroviral protein database.

Protein	Best reciprocal BLASTp Hit	Accession Number	Query Coverage	E-Value	% Identity
Gag	Gag polyprotein [Feline foamy virus]	AAC58530.1	95%	5 × 10^−33^	27%
Pol	Pol [Rhesus macaque simian foamy virus]	YP_009513242.1	96%	0.0 *	44%
Env	Env protein [Japanese macaque simian foamy virus]	YP_009508557.1	91%	1 × 10^−101^	28%

* as explicitly reported by the program.

## Supplementary Materials

The following are available online at https://www.mdpi.com/1999-4915/11/7/641/s1, Figure S1: ERV-Spuma-Spu consensus sequence, Figure S2: Recombination detection in *pol-env* alignment, first round, Figure S3: Recombination detection in *pol-env* alignment, second round, Table S1: tBLASTn using CoeEFV Pol as probe against the tuatara genome, Table S2: Merged Pol hits and reciprocal BLASTx results, Table S3: tBLASTn using CoeEFV Env as probe against the extended Pol hits, Table S4: foamy virus-like endogenous viral elements in other reptiles, Table S5: host evolutionary timescales, Table S6: foamy virus-host association, Data S1: alignment of ERV-Spuma-Spu sequences – virus body portion, Data S2: alignment of ERV-Spuma-Spu sequences – long terminal repeat portion, Data S3: ERV-Spuma-Spu consensus sequence, Data S4: *pol*-*env* alignment, Data S5: Env alignment, Data S6 Pol alignment.
