# Supplementary material for: The First Co-Opted Endogenous Foamy Viruses and the Evolutionary History of Reptilian Foamy Viruses
Source: Viruses. 2019 Jul 12;11(7):641. doi: 10.3390/v11070641 (PMC6669660; doi:10.3390/v11070641)
Supplement: Supplementary file 1 [file viruses-11-00641-s001.zip › viruses-512695-suppl/Figure S1 ERV-Spuma-Spu consensus sequence.pdf]

Figure S1 ERV-Spuma-Spu consensus sequence

```
     5'-LTR
   1 TGTCATGTATCCTGTATATAGGCATATATTTTTGACCATCTGTGTTTCAATATATTGGAAATCAGAATTAGCCTCTCTGTATGATGACTT

  91 CTTAAAAGAGCTGTCCTATAGTGACCTTTCTACTGAACTCTTGACCAGATACTGGAAAATCAGCCTGTATACTGGAATAGAAGATGTAAT

 181 CAAGTCTGTCTCTTAAAGAAATYAACATTTTCTGTTGACTAGWCTCAAGATACATGACTCCCAGGCAGGGCAGGAAAAGGCCTTGTTTGT

 271 TTGGAGTTTACTCATTGTTAATCCTATCTTAKATGATAGCAACCTGTTTAGTATGTATCAACAGGCTTCTTACTATGTATTTTGAGAAAA

 361 ACCCCTCCCTGTCAGCAGTTTTGACCTGACGTCATTTTTAGCTGACYTCCAAGCATCTTTCATTCTATAAGAAGGGAGAGGAAARATGGA

 451 TTTTTCAGAAAGCTTCCTTYCCTGAAGCTTTTCTCATCTTCCATCTGGCAGAATGGGGCAAAGGACTGGGTAATATGWGTATATCCTATA

 541 TGATGCATTGAGTCAACCATAGCAATAATACTTAGAGCATATGTTATATCTTATAGATGTTAAGATCTTTGAATTTGTAACAGAGCTGTT

 631 CTTTGAACAGAAATGTCTTTCTAAGTCATGTACTTTTAACTGACACTGTTTCTTTTGTACTATGTCTTTATTTGAACTTAAGTAAACTAT

 721 TGCTTCATATTTTTATAATATTCTTAAGTCTCATAGCYTCATTTATATAAATRAATTGTCTTTACTATACTTTGCTGAGCTGGCTGGCTT

 811 ATTAGCAAAGTGTAAACTCGAATTCCACATATTGGAAAAGAGCGCCCTATATCCAGACCAGATCATGTCAGGCCTGGACCACTAAGCGCA
                                                   PBS (tRNA-Lys)
 901 AATTCCTTTTGGGGAAACTGAGGCACAAYGCGACAATTGGCGCCCAAYGTGGGGCTCGAGACATAAGAATATATAATTAAGTACACTGGG

 991 GTGTTCACCAGACCGTTAACTACTTTTGTCCTCTTGATATATTCRAGTTTGTTAAGCTTAACTAAAAGAAAAGGATTTTTGTGCATTTTA
          ➔ Gag ORF start
Gag       M  A  I  Q  L  N  Q  V  P  Y  A  L  W  V  A  N  L  Q  N  V  T  V  R  D  G  D  D  Y  C
1081 TATCATGGCTATTCAATTAAACCAAGTGCCTTATGCACTATGGGTTGCTAATCTGCAAAATGTGACAGTCAGAGATGGAGATGATTATTG

Gag    L  E  I  R  N  G  E  W  G  I  G  H  R  F  L  I  V  S  F  E  A  N  D  A  G  V  I  V  S  M
1171 CTTAGAAATAAGAAATGGAGAATGGGGAATTGGACACAGATTTCTAATTGTTTCTTTTGAAGCAAATGATGCAGGTGTAATAGTTTCTAT

Gag    R  L  R  D  V  V  F  N  P  L  T  M  P  V  L  P  I  N  R  Q  D  L  N  L  M  A  G  I  V  V
1261 GAGACTAAGAGATGTAGTTTTTAATCCATTGACAATGCCTGTGTTACCTATCAATAGGCAAGATTTGAACCTAATGGCAGGGATAGTAGT

Gag    E  I  P  K  N  I  R  R  H  G  P  I  S  I  S  N  A  D  Y  I  S  G  S  G  Y  N  Y  T  P  A
1351 TGAAATTCCCAAAAATATAAGAAGGCATGGGCCTATTTCTATAAGTAATGCTGATTATATTTCTGGAAGTGGATATAATTATACTCCAGC

Gag    T  T  E  E  L  R  G  F  N  A  A  Q  W  E  Q  E  Y  T  L  L  R  R  E  Y  L  N  L  R  E  F
1441 TACCACTGAGGAATTGCGTGGCTTTAATGCAGCCCAGTGGGAACAGGAGTACACTCTCTTAAGRAGAGAATATTTAAATCTGAGAGAATT

Gag    A  R  S  N  V  G  R  Q  P  V  V  P  G  A  H  P  A  E  I  S  V  L  N  M  A  N  I  R  A  V
1531 TGCACGAAGTAATGTTGGAAGACAACCTGTTGTGCCAGGAGCACACCCAGCTGAAATTTCTGTCTTAAATATGGCAAATATACGGGCTGT

Gag    T  G  P  T  P  K  D  F  K  E  I  P  M  W  F  E  S  H  L  S  A  L  E  A  V  T  S  T  A  S
1621 AACTGGGCCCACTCCAAAAGATTTTAAAGAAATTCCTATGTGGTTTGAATCCCATTTATCTGCTTTAGAAGCTGTCACTTCCACAGCATC

Gag    P  L  Q  K  M  R  L  C  N  S  L  V  P  A  S  A  A  L  I  E  Q  E  C  N  D  W  E  S  V  L
1711 ACCATTGCAGAAGATGAGGTTATGCAATTCTCTAGTYCCAGCATCTGCAGCTTTAATAGAGCAGGAGTGTAATGATTGGGAAAGTGTATT

Gag    A  N  L  Y  V  K  T  H  G  Q  V  G  I  A  D  L  H  X  I  L  R  K  I  T  Q  E  Q  G  I  V
1801 GGCTAATTTATATGTAAAAACTCATGGACAAGTGGGCATAGCTGACTTACATRAAATTCTAAGAAAGATAACTCAAGAGCAAGGCATTGT

Gag    R  A  Y  G  V  G  M  K  F  L  S  N  H  D  L  I  W  G  I  L  K  T  L  C  K  G  D  V  L  K
1891 AAGGGCCTATGGGGTTGGAATGAAATTTCTCTCTAATCATGATTTGATATGGGGAATCCTTAAAACCCTCTGTAAAGGTGATGTATTGAA

Gag    A  A  I  Q  S  K  L  D  L  L  I  T  E  Q  E  K  I  R  S  F  P  K  I  V  Q  D  I  Y  K  T
1981 AGCTGCCATTCAATCTAAATTAGATTTGCTGATTACTGAGCAGGAAAAGATAAGGTCCTTCCCAAAAATTGTMCAGGATATTTATAAGAC

Gag    L  G  R  D  Y  L  G  D  N  P  N  R  K  N  L  E  Q  E  G  S  K  S  K  K  S  P  S  N  T  N
2071 TCTGGGTAGAGATTATTTGGGAGATAATCCAAATAGGAARAATCTTGAACAGGAAGGTTCTAAATCTAAGAAATCTCCCTCAAATACTAA

Gag    S  K  N  K  F  V  P  Q  N  S  Q  P  N  Y  R  G  K  G  G  K  N  Q  G  S  N  R  K  Q  F  Q
2161 TTCTAAAAATAAATTTGTTCCCCAGAATTCCCAGCCAAATTATAGAGGCAAAGGAGGGAAGAACCAAGGCTCAAATAGAAAGCAGTTCCA

Gag    A  Q  K  N  Q  D  S  E  N  P  E  G  A  A  T  Y  D  L  R  K  G  S  Q  F  P  H  R  D  F  D
2251 AGCTCAGAAAAACCAGGATTCTGAAAATCCAGAAGGTGCAGCTACATATGACTTAAGGAAAGGATCACAGTTTCCCCATAGAGACTTTGA
```

```
                                                    Gag ORF end ◆─┐
Gag    K  S  K  Q  D  F  N  S  R  G  G  F  R  G  N  K  *
 2341 TAAATCAAAGCAAGATTTTAATTCCAGAGGTGGATTTAGAGGAAATAAATGAGGTTTTAATAAAAGAGGAAACTTTCAGCCCTCTAGAGA
                                                         ┌─▶ Pol ORF start
Pol                                                        M  A  E  N  R  Q  A  Q  V  S  L
 2431 CTTTGATCAACTGACATCCTCACAGAAAGCTTCAGAAACTCCCTCTGTGACACAGGATGGCAGAGAACAGACAAGCTCAGGTCAGCCTTG

Pol   E  F  P  M  V  S  V  E  I  A  D  K  A  F  T  A  L  I  D  T  G  A  N  Q  S  C  I  Q  S  K
 2521 AATTTCCAATGGTCTCTGTAGAAATAGCAGATAAGGCTTTYACAGCTCTGATAGACACAGGGGCAAATCAATCTTGTATTCAATCAAAAT

Pol   C  L  P  H  V  Y  T  T  K  G  Q  C  I  V  E  S  Y  F  L  E  E  K  V  V  P  L  V  K  L  P
 2611 GTTTGCCACATGTATATACAACTAAAGGTCAATGTATAGTTGAGTCTTATTTTTTAGAAGAGAAAGTAGTACCTCTTGTAAAATTACCTG

Pol   V  Q  I  A  G  Q  C  L  E  I  E  F  A  I  C  D  F  T  K  H  D  V  V  I  A  H  E  R  V  K
 2701 TTCAGATAGCAGGACAATGTTTGGAAATTGAATTTGCAATATGTGATTTTACAAAACATGATGTTGTCATAGCTCATGAAAGGGTTAAGG

Pol   D  L  F  P  I  G  S  I  N  I  I  G  T  Q  D  N  R  G  E  Q  I  K  E  Q  I  A  S  A  D  C
 2791 ATTTGTTTCCAATAGGTTCAATAAACATAATTGGAACTCAAGATAATAGGGGTGAGCAAATCAAAGAGCAAATTGCATCAGCTGACTGTG

Pol   A  K  N  E  K  I  K  L  R  D  I  L  Y  S  L  K  P  Y  F  Q  Q  F  D  N  Q  I  G  H  R  K
 2881 CAAAAAATGAAAAAATAAAATTGAGAGATATTTTATACTCTCTGAAACCATATTTTCAGCAATTTGATAATCAAATTGGCCACAGGAAAA

Pol   I  K  P  H  D  L  S  V  K  T  Q  P  K  P  Q  K  Q  Y  P  I  N  K  A  A  I  N  D  I  Q  K
 2971 TAAAGCCTCATGATTTGTCTGTCAAAACACAGCCCAAACCGCAAAAGCAATATCCTATTAACAAAGCTGCAATTAATGATATTCAAAAGG

Pol   V  I  N  D  L  I  A  Q  G  A  L  I  R  Q  Y  S  S  M  N  T  P  V  Y  P  V  P  K  P  N  G
 3061 TAATTAATGATTTAATTGCACAAGGAGCATTGATTAGACAATATAGCTCCATGAATACTCCAGTATATCCAGTGCCTAAACCTAATGGGA

Pol   K  W  R  M  V  L  D  Y  R  A  L  N  R  V  S  P  S  F  N  V  Q  N  L  H  M  S  G  M  L  G
 3151 AATGGCGTATGGTATTAGACTACCGTGCCCTAAACAGGGTTTCACCCTCTTTTAATGTGCAAAACCTACACATGTCTGGCATGTTAGGAA

Pol   N  L  E  R  H  K  Y  K  T  T  L  D  L  S  N  G  F  W  A  H  P  I  R  E  Q  D  R  Y  L  T
 3241 ATTTGGAAAGGCACAAATACAAAACTACACTGGATTTATCTAATGGATTTTGGGCTCATCCTATTAGAGAACAAGACAGATATTTGACTG

Pol   A  F  S  W  Q  G  T  Q  Y  C  W  T  R  L  P  Q  G  Y  L  N  S  P  A  L  F  S  A  D  V  I
 3331 CTTTTAGTTGGCAGGGAACACAGTACTGTTGGACACGCCTTCCTCAAGGCTATTTAAATAGTCCAGCGTTGTTTTCAGCTGACGTAATTG

Pol   E  L  L  K  N  I  P  G  V  H  S  Y  M  D  D  I  Y  F  T  N  E  D  L  D  Q  H  L  A  T  L
 3421 AGCTTTTGAAAAATATTCCAGGTGTACATTCATATATGGATGACATCTACTTTACAAATGAAGATTTAGATCAACATTTAGCTACATTAA

Pol   K  Q  I  V  T  V  L  G  E  A  G  Y  I  I  N  L  K  K  S  Q  I  C  R  S  K  V  K  F  L  G
 3511 AGCAAATTGTCACTGTTTTGGGAGAAGCAGGATATATTATCAATCTGAAAAAATCACAGATTTGTAGAAGCAAAGTAAAGTTTCTTGGTT

Pol   F  Q  L  T  D  S  G  R  G  L  T  Q  E  F  K  E  K  L  L  T  L  Q  P  P  K  T  L  K  E  L
 3601 TCCAATTAACTGACTCTGGCAGAGGACTAACTCAGGAATTCAAAGAAAAACTGTTGACATTGCAACCTCCTAAAACTTTAAAAGAATTGC

Pol   Q  S  V  L  G  F  L  N  V  A  R  I  L  V  P  D  Y  A  Q  R  T  K  P  L  Y  N  L  I  P  L
 3691 AGTCAGTTTTAGGTTTTTTAAATGTTGCAAGAATATTGGTTCCTGACTATGCTCAGAGAACCAAACCCCTTTATAATCTAATTCCATTAG

Pol   A  S  K  G  N  F  W  T  L  E  A  Q  Q  T  L  D  N  L  I  T  L  I  N  Q  A  V  E  F  N  T
 3781 CAAGCAAAGGGAATTTTTGGACCTTGGAAGCTCAGCAAACATTGGATAATYTGATAACCTTAATTAACCAAGCTGTTGAATTTAACACAA

Pol   R  N  S  T  V  S  L  E  I  L  V  G  A  T  Q  K  G  G  F  A  S  Y  F  N  Y  G  E  S  K  P
 3871 GAAACAGCACAGTTTCCCTAGAAATTCTAGTTGGAGCCACACAGAAAGGAGGATTTGCTTCATATTTCAATTATGGAGAATCTAAACCTC

Pol   L  Q  Y  I  S  Y  V  F  S  N  A  E  Q  K  F  L  P  I  E  R  I  L  C  M  C  N  L  A  I  L
 3961 TGCAGTACATATCATATGTCTTTTCAAATGCAGAACAAAAGTTTTTGCCTATTGAAAGAATCTTATGTATGTGTAATCTTGCAATTTTAA

Pol   K  G  K  D  L  A  Q  G  Q  K  M  I  V  K  T  P  I  A  S  L  R  Q  V  K  K  G  S  I  P  N
 4051 AAGGTAAAGATCTTGCACAGGGCCAAAAGATGATAGTAAAAACTCCTATTGCCTCCTTAAGGCAGGTTAAAAAAGGATCAATACCCAATG

Pol   A  K  A  L  H  S  R  W  V  Q  W  M  S  H  F  E  N  P  Q  I  E  F  E  Y  V  E  P  S  N  D
 4141 CAAAAGCTCTTCAYAGCAGATGGGTACAATGGATGTCACATTTTGAAAATCCACAAATAGAATTTGAATATGTGGAACCAAGCAATGATT

Pol   L  E  N  L  P  A  F  T  I  E  P  V  S  S  K  N  H  T  P  D  K  P  L  H  E  Y  Q  K  V  I
 4231 TAGAAAATTTGCCAGCTTTTACTATTGAACCTGTATCTTCTAAAAATCATACCCCAGATAAACCTCTCCATGAATATCAAAAAGTTATCT

Pol   Y  T  D  G  S  A  M  S  C  K  Q  G  K  H  M  W  K  A  G  C  A  V  V  I  G  T  F  N  D  K
 4321 ATACTGATGGTTCAGCCATGTCATGTAAACAAGGCAAACACATGTGGAAAGCAGGTTGTGCTGTTGTAATTGGGACATTTAATGATAAAG

Pol   G  E  Y  H  M  S  D  S  I  Q  M  P  L  G  N  N  T  A  Q  Y  A  E  L  M  A  V  H  K  A  I
```

```
 4411 GGGAATATCATATGTCTGACAGTATTCAAATGCCTTTAGGAAAYAATACTGCGCAATACGCTGAGTTGATGGCAGTACATAAAGCAATAG

Pol   E  I  S  P  P  D  A  T  V  L  I  C  T  D  S  F  Y  I  A  R  G  I  N  E  E  L  S  I  W  R
 4501 AGATCTCTCCTCCTGATGCAACTGTTCTCATTTGTACTGATTCCTTCTATATAGCTAGAGGAATAAATGAAGAATTGTCGATCTGGCGGT

Pol   S  N  G  F  L  D  N  K  R  K  P  L  K  H  A  H  R  W  Q  K  L  A  T  L  L  D  D  K  P  L
 4591 CTAATGGTTTTCTAGATAATAAACGAAAACCTTTGAAACATGCCCATAGATGGCAAAAATTAGCAACACTTTTGGATGACAAGCCATTGA

Pol   I  T  V  M  H  V  P  G  H  S  K  Y  G  S  H  V  N  G  N  T  L  V  D  L  L  A  K  E  A  M
 4681 TTACTGTAATGCACGTGCCAGGCCATTCCAAATATGGATCTCATGTTAACGGGAACACCCTTGTAGACTTACTGGCAAAGGAAGCAATGA

Pol   K  A  S  S  V  C  V  L  T  R  S  Q  V  K  K  C  L  D  G  E  L  T  Q  C  I  S  P  D  S  I
 4771 AAGCATCCTCAGTCTGTGTACTTACCCGGTCACAAGTTAAAAAATGCTTGGATGGGGAATTGACTCAATGCATCAGCCCAGATTCCATTA

Pol   N  P  K  G  Y  P  S  A  Y  D  Y  A  L  K  D  G  K  C  V  V  T  F  T  N  G  E  K  R  V  I
 4861 ACCCTAAGGGTTACCCTTCTGCCTATGATTATGCCCTAAAAGATGGGAAGTGTGTTGTAACATTCACTAACGGGGAGAAACGTGTAATAC

Pol   P  P  V  D  T  R  P  N  L  I  Q  E  A  H  N  S  L  G  H  V  H  Q  G  V  N  A  T  V  E  S
 4951 CCCCTGTTGACACAAGACCTAACTTAATACAAGAGGCTCACAATAGTCTTGGGCATGTTCACCAAGGTGTTAATGCCACAGTTGAATCTT

Pol   L  Q  H  S  Y  W  W  P  G  L  R  K  Q  V  Q  X  X  L  A  Q  C  E  P  C  L  R  T  N  P  G
 5041 TACAGCATTCTTATTGGTGGCCTGGACTGCGCAAACAAGTCCAAYGGCRTTTGGCCCAGTGTGAACCTTGTCTAAGAACAAACCCTGGTC

Pol   P  V  T  R  P  P  Y  L  K  N  P  K  P  L  S  P  F  D  K  V  Y  M  D  Y  I  G  P  L  P  P
 5131 CAGTTACCAGACCACCTTACTTAAAAAATCCTAAGCCTCTGTCTCCTTTTGATAAGGTGTATATGGATTATATTGGTCCTTTGCCACCTT

Pol   S  H  G  H  N  H  C  L  V  I  V  D  A  C  T  G  F  V  W  I  Y  P  T  R  D  Q  S  A  S  T
 5221 CCCATGGGCATAATCACTGTTTGGTTATTGTTGATGCTTGTACTGGTTTTGTATGGATTTACCCCACACGAGATCAATCTGCCTCTACCA

Pol   T  V  K  T  L  T  S  F  I  S  L  G  L  P  R  I  L  H  S  D  K  G  G  A  F  T  S  H  Q  M
 5311 CTGTTAAAACTCTCACCTCCTTCATTTCGCTTGGGCTTCCACGTATCCTACACTCTGACAAGGGAGGTGCCTTCACCTCTCACCAAATGC

Pol   Q  S  F  A  K  S  F  G  I  V  L  E  Y  S  T  P  Y  H  P  Q  S  A  G  V  V  E  R  K  N  G
 5401 AAAGTTTTGCAAAGAGTTTTGGGATAGTGTTGGAATATAGCACACCTTATCACCCCCAAAGTGCTGGGGTTGTGGAAAGGAAAAATGGAG

Pol   E  I  K  R  A  L  T  K  L  L  V  G  R  S  R  Q  W  Y  S  L  L  P  L  V  Q  L  G  L  N  N
 5491 AGATAAAACGAGCTTTAACAAAGCTATTGGTGGGGAGATCCCGGCAGTGGTATTCTTTGCTTCCTCTGGTACAGCTTGGACTTAATAACC

Pol   L  P  R  S  D  C  H  L  T  P  Y  K  L  L  F  A  K  D  M  T  T  P  L  E  Q  L  A  L  S  S
 5581 TTCCTAGGAGTGACTGTCACTTAACTCCTTATAAATTGCTATTTGCAAAGGACATGACCACTCCTTTAGAACAACTGGCTTTATCTTCTC

Pol   P  I  S  R  Q  E  Q  L  A  L  I  D  E  L  R  A  E  L  A  S  P  Q  P  N  T  D  I  N  S  V
 5671 CTATTTCTCGACAGGAGCAGTTGGCACTGATAGATGAATTGAGAGCAGAACTGGCTTCTCCCCAGCCTAACACTGATATTAATTCTGTTC

Pol   P  R  V  W  I  P  Q  E  G  Q  L  V  Q  E  K  A  V  L  K  D  P  S  L  R  P  R  W  K  K  P
 5761 CTCGAGTTTGGATTCCACAGGAAGGCCAGCTGGTACAGGAAAAGGCTGTTTTAAAGGATCCTTCCTTAAGGCCTCGCTGGAAAAAGCCTG

Pol   V  P  I  L  K  V  L  S  P  R  A  V  E  I  Q  T  S  P  G  N  S  K  I  V  S  I  D  N  L  K
 5851 TACCTATTCTCAAAGTTTTGAGTCCCCGAGCAGTAGAAATTCAGACATCACCAGGTAATTCGAAAATTGTGTCTATTGACAATTTAAARC

                        Env ORF start                                    Pol ORF end
Env                     M  V  R  V  M  M  D  Y  C  Q  I  P  L  Q  P  Q  P  L  P  P  T  A  P  D  Y
Pol   R  T  P  I  H  Y  G  Q  G  N  D  G  L  L  S  N  S  T  S  T  P  T  S  P  T  N  S  T  *
 5941 GTACTCCCATCCACTATGGTCAGGGTAATGATGGATTATTGTCAAATTCCACTTCAACCCCAACCTCTCCCACCAACAGCACCTGATTAC

Env    S  L  A  M  D  E  V  S  Q  K  A  P  L  P  Q  P  S  L  R  Q  Y  F  W  P  R  Y  R  R  F  T
 6031 TCTCTTGCTATGGATGAAGTGTCTCAAAAGGCACCTCTGCCACAGCCAAGCCTCAGACAATATTTTTGGCCTAGGTACCGAAGATTTACA

Env    S  C  F  L  W  S  I  F  V  F  F  C  I  T  I  I  T  F  T  I  V  F  S  V  L  R  L  T  W  A
 6121 TCTTGTTTTTTATGGAGCATTTTTGTTTTCTTTTGCATAACTATTATAACTTTTACTATTGTTTTTTCAGTCCTTCGTTTAACATGGGCT

Env    H  T  V  S  L  P  A  T  P  I  H  W  N  L  S  E  A  H  N  G  S  V  V  Y  R  R  M  H  N  A
 6211 CATACTGTTTCAYTACCCGCTACTCCAATTCATTGGAACCTCTCCGAGGCACACAATGGTAGTGTTGTGTATCGAAGGATGCATAATGCC

Env    R  P  T  R  A  L  H  I  E  V  V  P  V  H  V  E  T  A  G  I  P  F  G  I  I  H  N  P  F  P
 6301 AGACCTACACGTGCTCTTCATATAGAGGTTGTTCCAGTACACGTGGAGACTGCAGGTATTCCATTTGGGATCATACATAATCCCTTTCCT

Env    K  P  I  V  S  Q  R  S  E  L  L  V  P  F  T  L  N  I  D  T  R  A  L  A  Y  C  S  G  L  F
 6391 AAGCCTATTGTTTCCCAACGAAGTGAACTGTTGGTTCCTTTTACTTTAAACATAGACACCAGAGCGCTAGCTTATTGTTCTGGCTTGTTC

Env    S  K  E  A  N  T  H  L  A  K  T  I  E  E  D  L  R  D  L  D  S  R  N  A  H  F  L  V  P  G
 6481 TCAAAAGAAGCTAACACACATCTTGCAAAGACTATAGAAGAAGACTTGCGAGATTTGGACAGTAGAAATGCACATTTTCTTGTTCCAGGT
```

```
Env    T  D  P  W  H  Q  T  S  Y  A  D  K  M  C  F  A  S  Y  G  H  C  Y  F  V  S  Y  G  K  P  R
6571  ACAGATCCTTGGCATCAAACAAGCTATGCTGATAAAATGTGCTTTGCTTCCTATGGACATTGTTATTTTGTTTCCTATGGAAACCGAGG

Env    K  W  P  R  P  H  V  Y  A  D  H  C  D  R  P  Q  F  W  T  D  I  K  T  A  T  Q  G  L  P  Q
6661  AAATGGCCAAGACCTCATGTGTATGCAGATCACTGTGACAGACCTCAATTTTGGACTGACATTAAAACTGCAACACAAGGCCTGCCTCAG

Env    W  Y  L  A  I  D  D  F  S  D  H  L  R  Y  A  K  Q  Q  R  S  G  G  E  D  X  E  Y  R  V  P
6751  TGGTACCTAGCTATTGATGATTTTTCTGATCACTTGCGCTATGCTAAGCAGCAACGTTCTGGAGGTGAAGACCRTGAATACAGAGTTCCA

Env    G  G  Q  L  P  Y  T  G  A  I  F  C  T  S  F  L  Y  N  T  S  W  W  D  E  S  N  L  S  V  D
6841  GGTGGCCAGTTGCCCTATACTGGAGCAATTTTTTGCACATCTTTTTTATATAATACCTCTTGGTGGGATGAATCAAATTTATCTGTGGAT

Env    G  S  L  E  L  K  S  I  L  T  S  C  L  A  N  S  T  T  G  K  L  K  P  K  C  L  S  S  Q  W
6931  GGTTCTTTAGAATTAAAGTCCATTTTGACTTCTTGTCTTGCAAATTCTACTACTGGGAAGCTCAAACCTAAGTGTTTATCCTCTCAATGG

Env    H  D  N  G  A  N  E  M  F  I  G  V  T  G  T  S  F  C  D  I  P  R  Y  P  I  F  L  N  R  S
7021  CATGATAATGGTGCTAATGAGATGTTTATTGGTGTTACTGGGACTTCATTTTGTGATATCCCTAGGTATCCTATTTTTCTCAATAGATCT

Env    E  S  I  V  S  C  K  S  T  F  V  N  P  Q  Q  Q  P  L  E  C  G  N  D  K  A  L  A  A  K  G
7111  GAGAGTATTGTATCTTGTAAGAGTACCTTTGTTAATCCTCAACAACAGCCCCTTGAATGTGGCAATGATAAAGCTCTCGCTGCTAAGGGT

Env    L  H  S  W  N  C  G  P  C  S  V  N  I  T  A  N  L  M  G  N  Y  T  A  K  E  R  A  S  L  G
7201  TTACATTCATGGAATTGTGGSCCATGCAGTGTTAATATTACTGCRAATCTCATGGGTAATTATACAGCCAAAGAGAGGGCGAGTTTGGGA

Env    N  K  R  W  F  N  L  I  Q  G  P  L  F  V  N  A  T  P  F  F  A  D  N  Y  A  I  Y  S  L  Y
7291  AATAAGCGTTGGTTTAACTTGATACAAGGTCCATTATTTGTCAACGCTACTCCATTTTTTGCTGATAATTATGCCATTTATTCGCTGTAT

Env    Q  K  C  K  T  L  S  E  K  H  S  L  F  S  V  L  Q  A  L  E  E  F  I  M  V  P  Q  E  N  E
7381  CAGAAATGTAAAACCTTATCTGAAAAACATTCTTTATTTTCAGTGCTTCAAGCATTAGAAGAATTTATCATGGTACCTCAGGAAAATGAA

Env    D  Y  P  C  T  H  S  C  I  N  A  S  L  L  Q  M  N  P  K  R  A  I  W  G  T  N  K  T  L  N
7471  GATTATCCTTGTACACATTCTTGTATTAATGCTTCTTTATTACAGATGAACCCCAAAAGGGCGATATGGGGCACAAATAAAACTCTTAAT

Env    D  I  H  V  L  A  T  P  D  D  T  X  E  S  F  P  S  S  T  Y  K  S  R  K  I  L  Q  V  E  S
7561  GATATTCATGTTTTAGCAACACCTGATGATACTAYAGAGAGTTTTCCTTCTTCCACTTATAAATCTAGGAAGATTTTACAAGTAGAATCT

Env    L  N  N  A  Q  I  F  R  K  T  N  F  L  L  A  K  S  M  E  K  I  S  R  L  Q  D  A  N  N  I
7651  TTGAATAATGCACAAATATTTAGAAAAACCAATTTCTTGCTCGCAAAATCTATGGAAAAGATTTCTAGATTGCAGGATGCCAATAATATA

Env    N  L  R  N  G  V  Y  L  V  K  D  A  L  T  Q  V  A  L  I  V  K  H  D  L  A  V  L  S  D  E
7741  AATTTAAGAAATGGAGTTTATTTGGTCAAGGATGCTTTAACTCAAGTGGCCTTGATAGTCAAACATGATTTGGCAGTTTTRTCTGATGAA

Env    L  I  M  E  I  I  V  T  X  L  Q  K  I  I  F  S  L  S  N  G  H  V  P  W  T  I  V  N  T  T
7831  TTGATTATGGAAATTATTGTCACTCRGTTGCAAAAAATTATTTTTTCTTTAAGCAATGGGCACGTTCCCTGGACTATTGTTAATACTACT

Env    E  I  Q  Q  S  L  S  L  S  D  E  N  M  R  I  L  Q  H  T  A  R  A  L  I  F  N  M  K  P  L
7921  GAGATTCAGCAATCCTTATCTTTAAGTGATGAGAACATGCGAATCYTACAGCACACAGCTCGTGCTTTGATTTTTAACATGAAACCATTA

Env    P  D  S  V  L  T  D  P  L  N  M  W  Y  I  P  T  T  R  W  N  I  H  F  L  I  H  L  K  I  P
8011  CCAGATTCAGTTCTTACTGACCCCCTAAATATGTGGTATATTCCTACTACTCGATGGAATATTCATTTTTTAATTCATTTGAAAATTCCT

Env    S  M  V  Y  L  T  N  W  N  I  L  N  M  G  F  L  V  A  T  G  T  N  I  A  H  A  H  M  K  L
8101  AGTATGGTTTATTTGACTAATTGGAATATTTTGAATATGGGTTTTTTAGTAGCTACTGGAACAAATATTGCTCATGCTCATATGAAACTA

Env    P  Y  E  Y  V  S  T  D  V  F  G  N  I  T  Y  L  H  T  D  E  C  Q  D  F  G  Y  L  F  C  K
8191  CCATATGAATATGTCTCCACAGATGTTTTTGGAAACATAACATACTTACATACTGACGAATGTCAAGATTTTGGATATTTGTTTTGCAAR

Env    Q  I  T  H  V  S  P  C  G  L  V  P  P  S  T  T  S  C  P  I  R  I  Q  S  S  N  I  S  F  V
8281  CAAATTACACATGTTTCCCCTTGTGGACTGGTTCCACCTAGTACTACTTCTTGTCCTATTAGGATTCAATCAAGTAATATTTCATTTGTT

Env    F  I  D  S  L  T  N  G  S  Y  I  I  L  A  G  K  S  E  C  N  I  P  A  L  Q  P  S  I  V  T
8371  TTTATTGATTCCCTTACAAATGGATCATATATAATYCTTGCTGGAAAATCTGAATGCAATATTCCAGCTTTGCAACCWTCTATCGTAACA

Env    V  N  S  T  I  T  C  Y  G  R  Q  L  F  P  P  P  N  L  G  S  M  H  S  Q  V  T  F  F  V  P
8461  GTGAATAGCACAATCACTTGTTATGGCAGACAACTTTTTCCACCTCCTAATTTGGGATCAATGCATTCTCAGGTTACATTTTTTGTACCT

Env    H  F  S  L  Q  F  P  L  L  T  G  I  I  A  K  L  Q  R  S  E  I  T  L  F  N  T  H  D  A  I
8551  CACTTTTCTTTGCAATTTCCATTATTAACTGGCATCATAGCTAAATTGCAAAGGTCAGAAATTACTCTTTTTAATACACATGATGCAATT

Env    E  D  I  L  Q  E  V  K  Q  L  L  L  R  I  D  I  H  E  G  D  F  P  L  W  L  N  R  L  A  X
```

8641 GAAGATATCTTGCAGGAAGTRAAGCAACTACTWYTAAGGATTGATATCCATGAAGGTGATTTTCCTCTATGGCTCAATAGATTGGCTWCA

Env A V S A A W P S L A N M A N S I V H A X T S I G T S I L G T

8731 GCTGTTTCTGCAGCCTGGCCTTCTTTAGCTAACATGGCTAATTCCATTGTTCACGCTGYTACTTCCATTGGTACTTCAATTTTAGGAACG

Env G L Q I I T Y L K P V L I A I V L I V L L I I V I K I F R F

8821 GGTTTACAAATTATTACCTACCTCAAACCAGTTTTAATTGCTATAGTGTTGATTGTATTATTAATCATAGTTATTAAGATTTTTAGGTTC

Env ORF end

Env F X G L R L K K V S A S K E D *

8911 TTCTMTGGACTGCGTCTGAAAAAAGTATCAGCCTCCAAAGAAGATTGATGTCTCTTCCCTCACMACTTGTGAAGAAGATACCAGTGCTTT

9001 GTACCCTCVCTGATGTCTTCCTGAAGACTTGAAAGACTGGACATTTGTTGTTAATTTTTCTTTCTTGTWTTGTATACATTCTTGAAACAC

3′-LTR

9091 ATTCTCGCCAAAGAGAGGG**TG**TCATGTATCCTGTATATAGGCATATATTTTTGACCATCTGTGTTTCAATATATTGGAAATCAGAATTAG

9181 CCTCTCTGTATGATGACTTCTTAAAAGAGCTGTCCTATAGTGACCTTTCTACTGAACTCTTGACCAGATACTGGAAAATCAGCCTGTATA

9271 CTGGAATAGAAGATGTAATCAAGTCTGTCTCTTAAAGAAATYAACATTTTCTGTTGACTAGWCTCAAGATACATGACTCCCAGGCAGGGC

9361 AGGAAAAGGCCTTGTTTGTTTGGAGTTTACTCATTGTTAATCCTATCTTAKATGATAGCAACCTGTTTAGTATGTATCAACAGGCTTCTT

9451 ACTATGTATTTTGAGAAAAACCCCTCCCTGTCAGCAGTTTTGACCTGACGTCATTTTTAGCTGACYTCCAAGCATCTTTCATTCTATAAG

9541 AAGGGAGAGGAAARATGGATTTTTCAGAAAGCTTCCTTYCCTGAAGCTTTTCTCATCTTCCATCTGGCAGAATGGGGCAAAGGACTGGGT

9631 AATATGWGTATATCCTATATGATGCATTGAGTCAACCATAGCAATAATACTTAGAGCATATGTTATATCTTATAGATGTTAAGATCTTTG

9721 AATTTGTAACAGAGCTGTTCTTTGAACAGAAATGTCTTTCTAAGTCATGTACTTTTAACTGACACTGTTTCTTTTGTACTATGTCTTTAT

9811 TTGAACTTAAGTAAACTATTGCTTCATATTTTTATAATATTCTTAAGTCTCATAGCYTCATTTATATAAATRAATTGTCTTTACTATACT

9901 TTGCTGAGCTGGCTGGCTTATTAGCAAAGTGTAAACTCGAATTCCACATATTGGAAAAGAGCGCCCTATATCCAGACCAGATCATGTCAG

9991 GCCTGGACCACTAAGCGCAAATTCCTTTTGGGGAAACTGAGGCACAAYGCGA**CA**
