# Supplementary figures and images for: The First Co-Opted Endogenous Foamy Viruses and the Evolutionary History of Reptilian Foamy Viruses
Source: Viruses. 2019 Jul 12;11(7):641. doi: 10.3390/v11070641 (PMC6669660; doi:10.3390/v11070641)

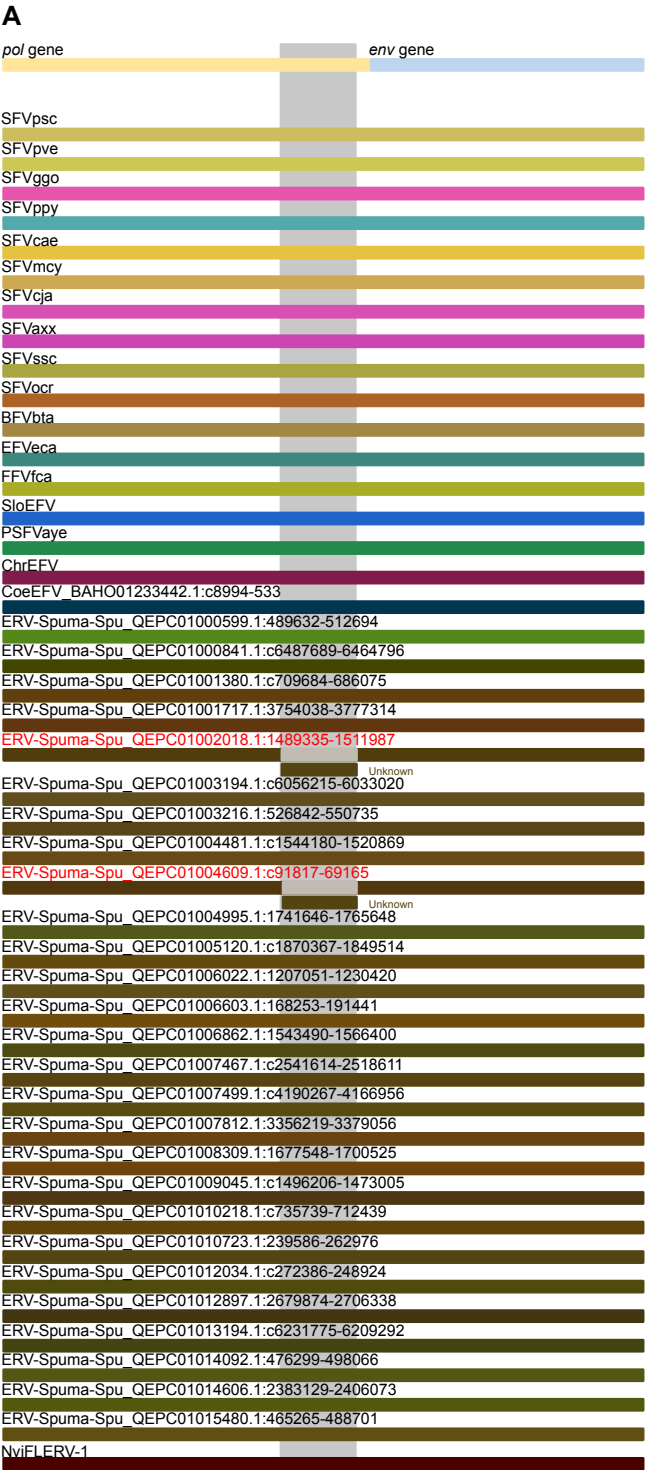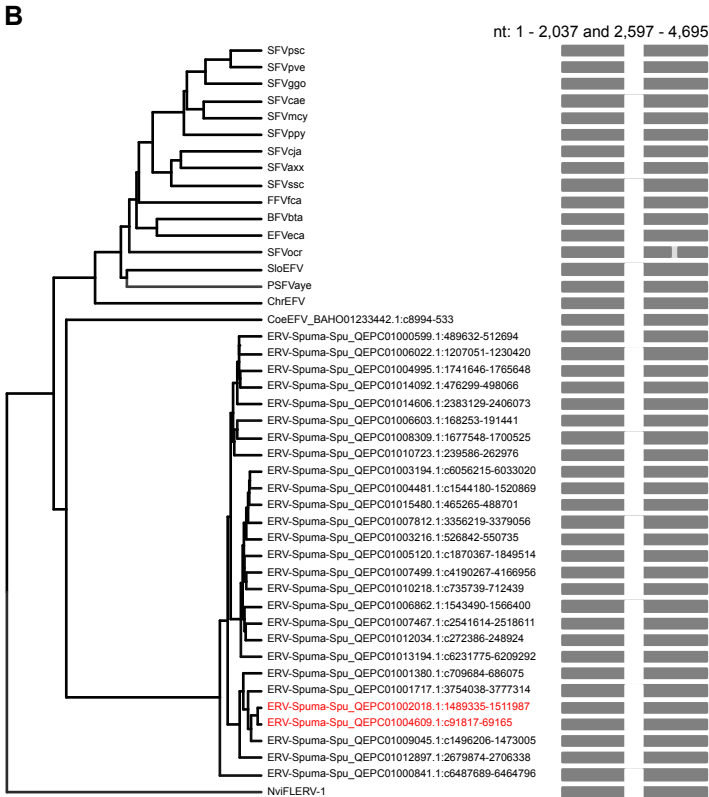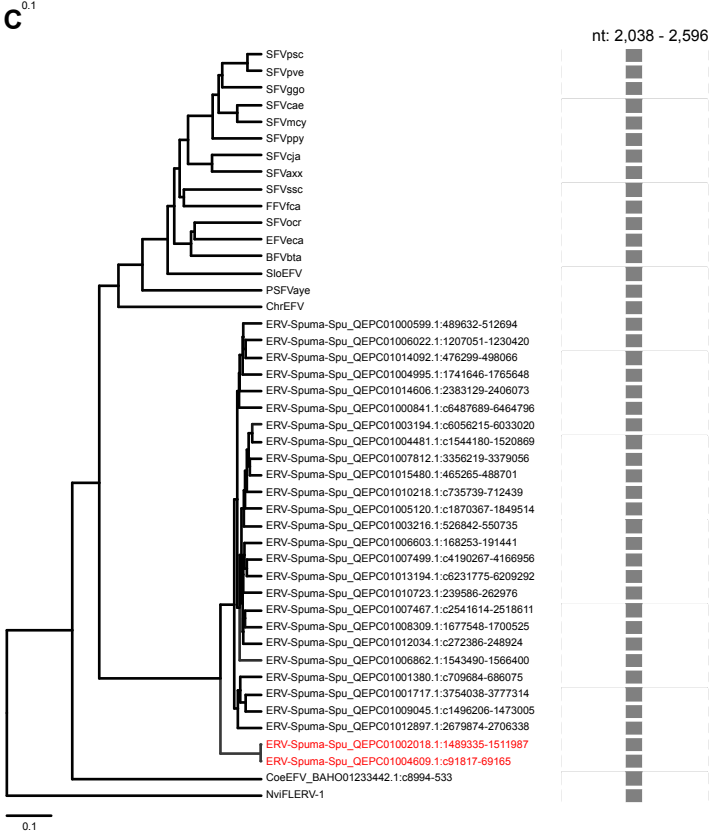

Supplement: Supplementary file 1 [file viruses-11-00641-s001.zip › viruses-512695-suppl/Figure S2 Recombination detection in pol-env alignment, first round.pdf]

**A**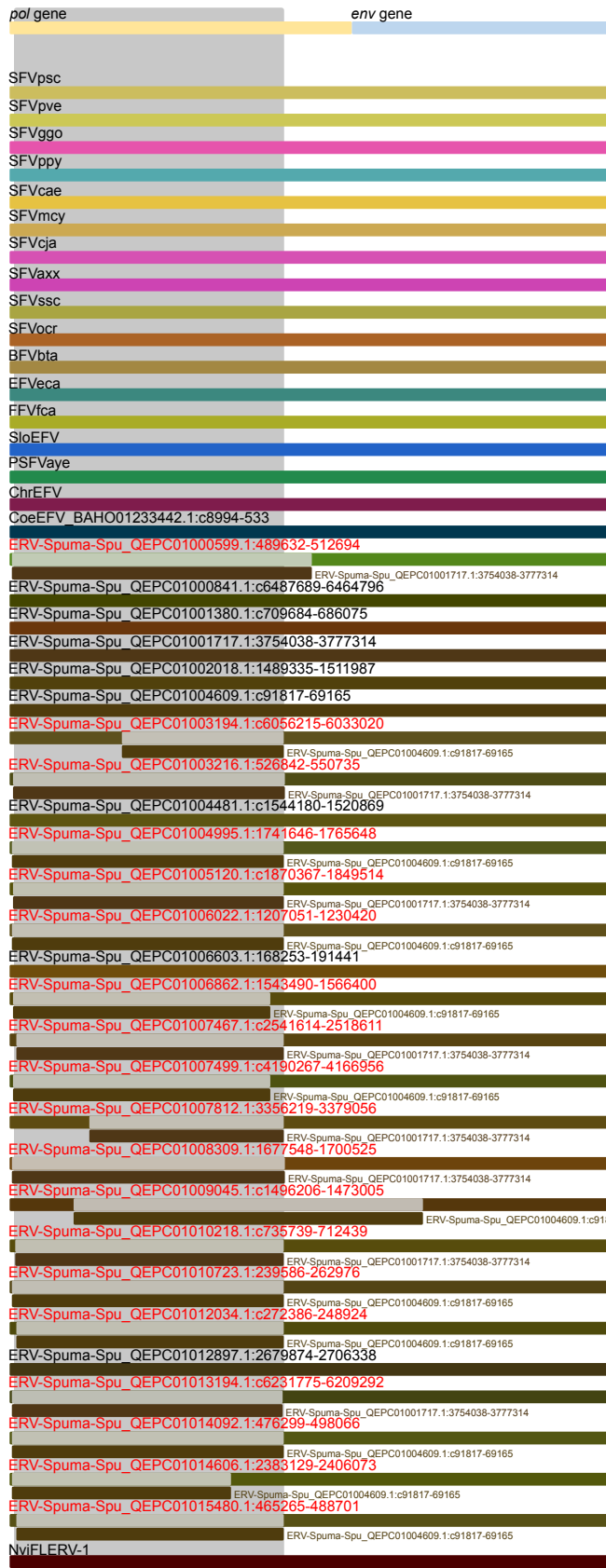**B**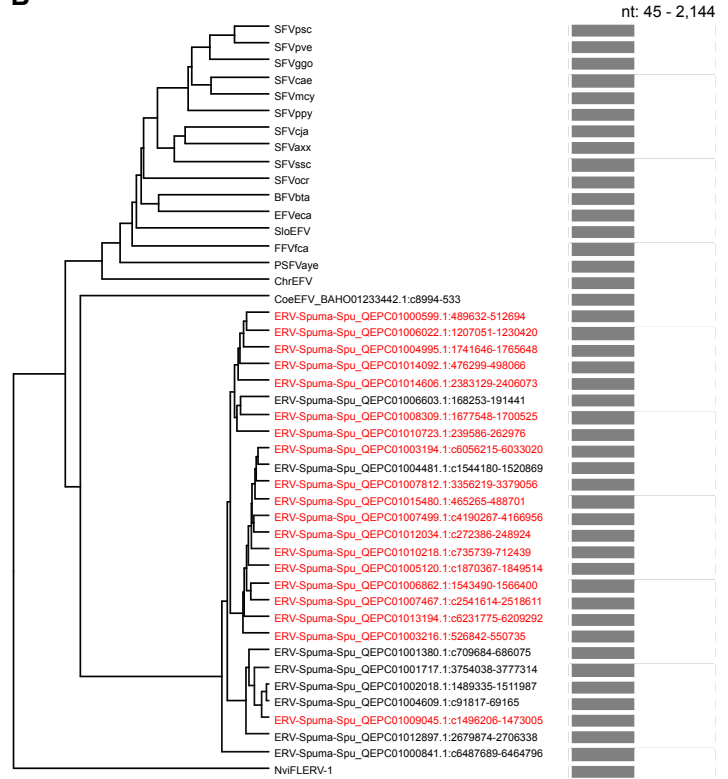**C**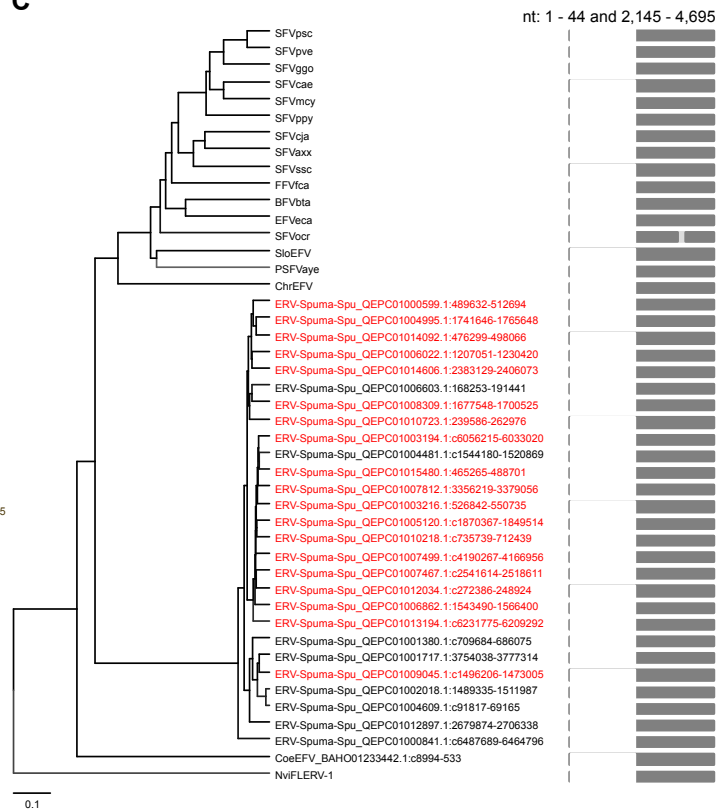

Supplement: Supplementary file 1 [file viruses-11-00641-s001.zip › viruses-512695-suppl/Figure S3 Recombination detection in pol-env alignment, second round.pdf]
